# Small extracellular vesicles released from germinated kiwi pollen (pollensomes) present characteristics similar to mammalian exosomes and carry a plant homolog of ALIX

**DOI:** 10.3389/fpls.2023.1090026

**Published:** 2023-01-25

**Authors:** Chiara Suanno, Elisa Tonoli, Enzo Fornari, Maria P. Savoca, Iris Aloisi, Luigi Parrotta, Claudia Faleri, Giampiero Cai, Clare Coveney, David J. Boocock, Elisabetta A. M. Verderio, Stefano Del Duca

**Affiliations:** ^1^ University of Bologna, Department of Biological, Geological, and Environmental Sciences, Bologna, Italy; ^2^ Nottingham Trent University, Interdisciplinary Biomedical Research Centre, School of Science and Technology, Nottingham, United Kingdom; ^3^ Chrysalis Health & Beauty Creation House, Nottingham, United Kingdom; ^4^ University of Bologna, Interdepartmental Centre for Agri-Food Industrial Research, Cesena, Italy; ^5^ University of Siena, Department of Life Sciences, Siena, Italy; ^6^ Nottingham Trent University, Department of Biosciences, Centre for Health, Ageing and Understanding Disease (CHAUD), School of Science and Technology, Nottingham, United Kingdom; ^7^ Nottingham Trent University, John van Geest Cancer Research Centre, Centre for Health, Ageing and Understanding Disease (CHAUD), School of Science and Technology, Nottingham, United Kingdom

**Keywords:** pollensomes, nanovesicles, pollen tube, pollination, exosomes, signaling, ALIX, EVs

## Abstract

**Introduction:**

In the last decade, it has been discovered that allergen-bearing extracellular nanovesicles, termed “pollensomes”, are released by pollen during germination. These extracellular vesicles (EVs) may play an important role in pollen-pistil interaction during fertilization, stabilizing the secreted bioactive molecules and allowing long-distance signaling. However, the molecular composition and the biological role of these EVs are still unclear. The present study had two main aims: (I) to clarify whether pollen germination is needed to release pollensomes, or if they can be secreted also in high humidity conditions; and (II) to investigate the molecular features of pollensomes following the most recent guidelines for EVs isolation and identification.

**Methods:**

To do so, pollensomes were isolated from hydrated and germinated kiwi (*Actinidia chinensis* Planch.) pollen, and characterized using imaging techniques, immunoblotting, and proteomics.

**Results:**

These analyses revealed that only germinated kiwi pollen released detectable concentrations of nanoparticles compatible with small EVs for shape and protein content. Moreover, a plant homolog of ALIX, which is a well-recognized and accepted marker of small EVs and exosomes in mammals, was found in pollensomes.

**Discussion:**

The presence of this protein, along with other proteins involved in endocytosis, is consistent with the hypothesis that pollensomes could comprehend a prominent subpopulation of plant exosome-like vesicles.

## 1 Introduction

It is widely known that protein secretion is needed for pollen-pistil communication during spermatophyte sexual reproduction, from the species-specific recognition, to the self-compatibility or incompatibility, to the pollen tube elongation (Cheung and Wu, 2008; Hafidh et al., 2014; Mandrone et al., 2019). However, proof that extracellular vesicles (EVs) are involved in such communication has been found only in the last two decades. Initially, Grote and colleagues demonstrated that allergenic pollen, when hydrated in rainwater, can release nanoparticles bearing pollen allergens (Grote et al., 2000; Grote et al., 2003). In 2014, Prado and colleagues proved that allergen-bearing nanoparticles released by germinated pollen grains are extracellular nanovesicles, with diameter ranging from 28 to 60 nm, which they named “pollensomes” (Prado et al., 2014; Prado et al., 2015). Since pollensomes were isolated using a protocol designed for mammalian exosomes, and because they were comparable in size with known exosomes, the researchers speculated that pollensomes could be plant exosomes (or small EVs, according with recent nomenclature) (Prado et al., 2014). EVs are a relatively new concept in biology and especially in plant science (Théry et al., 2018). They were first discovered in mammals and the bulk of knowledge about them derives from the study of mammalian cells, but they have also been described in other animals, yeasts, and plants (Rutter and Innes, 2017; Kurian et al., 2021). Exosomes can be defined as small EVs between 30 and 200 nm in diameter, with a lipidic double-layered membrane and endocytic origins. These features distinguish them from other known extracellular vesicles: nanovesicles derived by exocyst-positive organelles (EXPOs) have single-layered lipidic membranes (Wang et al., 2010), while extracellular microvesicles and apoptotic bodies are on average larger in size (50-1000 nm and 500-2000 nm, respectively) and originate by outward budding from the plasma membrane (Kurian et al., 2021), a phenomenon that has not been demonstrated for plants to date (Woith et al., 2021). Exosomes instead have a peculiar biogenesis, since they are produced by invagination of the late endosome membrane. At this first stage, exosomes are called intraluminal vesicles (ILVs), and the organelle including them is called multivesicular body (MVB). The membrane of the MVB then fuses to the plasma membrane, releasing the ILVs in the extracellular environment as exosomes (Johnstone, 2006; Javeed and Mukhopadhyay, 2017). While exosomes are known to mediate cellular signaling and several other biological functions in mammals (Javeed and Mukhopadhyay, 2017), their role in plants is yet to be fully understood. It is however ascertained that plant exosomes are involved in stress responses and defense signaling, and they may also mediate intercellular communication (An et al., 2007; Hansen and Nielsen, 2017; Rutter and Innes, 2017; Woith et al., 2021). Moreover, there is evidence that stigmatic papillae of Brassica napus L. secrete exosomes to communicate with pollen during pollen hydration and pollen tube entry (Goring, 2017). It is reasonable to wonder whether this type of communication might be adopted by the pollen too, since exosomes are known to enhance the effectiveness of signaling by protecting their cargo from degradation in the extracellular environment (Akuma et al., 2019).

According to recent literature, EVs can be defined exosomes if all the following criteria are met: (I) they can be isolated by ultracentrifugation at 100000 × g, (II) their dimensions fall within the accepted range for exosomes (30-200 nm), (III) they are released by MVBs, (IV) they contain accepted molecular markers for exosomes (Gurung et al., 2021; Kurian et al., 2021). Throughout the years, several molecular markers have been used to define mammalian exosomes, such as ALIX (Apoptosis-Linked gene-2 Interacting protein X), Tsg101 (Tumor Susceptibility Gene 101), tetraspanins (CD81, CD63, CD9), and flotillin; while for plant exosomes no universal molecular marker has been described yet, although some attempts have been made (Regente et al., 2009; Hafidh et al., 2016; Rutter and Innes, 2017; Woith et al., 2021). However, in the last years the International Society for Extracellular Vesicles has pointed out that proteins commonly regarded as molecular markers for exosomes are also enriched in small EVs of various origins, thus their presence cannot be correlated to a specific EVs subtype (Théry et al., 2018). For this reason, to date it is not possible to describe small EVs as exosomes without certain proof of their biogenesis.

The aim of this study was to deepen the knowledge on pollensomes, assessing (I) whether they could be secreted also by non-germinated pollen, under high humidity conditions; and (II) if they could be considered plant exosomes-like vesicles, i.e. if they met the most recent criteria to be defined small EVs, while having a protein content compatible with an endocytic origin. In particular, the presence of plant homologs of ALIX in pollensomes was investigated, since ALIX domain Bro1 is highly preserved in the evolution of eukaryotic organisms, and Bro1 domain-containing proteins are present in yeasts and plants, where they are known to be involved in the formation of ILVs and in the sorting of their cargo (Bissig and Gruenberg, 2014; Kalinowska et al., 2015; García-León and Rubio, 2020). In the present study, kiwi (Actinidia chinensis Planch.) pollen was used to investigate both the conditions that stimulate pollensomes release and the molecular content of these EVs, using a combination of imaging techniques, immunoblotting, and proteomic analysis that is required to gain insight on and to formally define small EVs.

## 2 Materials and methods

### 2.1 Plant material

Kiwi pollen has been chosen as a model for this study because it is easily available, it shows a high in vitro germination rate in only two hours, and it can be stored for several years without a significant decrease in viability and germinability. Kiwi pollen used in these experiments was purchased in 2019 from Azienda Agricola Tabanelli Pierino, Mirko e C. (Castel Bolognese, Bologna, Italy). Pollen was then dried and stored at -20°C.

### 2.2 Sample treatment

Each sample was made of 10 mg dry pollen for particle tracking analysis, atomic force microscopy, immunofluorescence, and immunogold. For FM4-64™ staining, samples were made of 20 mg dry pollen for each treatment, whereas 40 mg dry pollen samples were used for total proteomics and Western blot analysis. All the samples were initially rehydrated for 30 min at 30°C in a humid chamber with 100% relative humidity, and their viability was checked by MTT assay (Paris et al., 2017). Pollen was then resuspended in germination medium (10% sucrose, 324 µM H_3_BO_3_, 1.27 mM Ca(NO_3_)_2_) at concentrations of 1 mg/mL, and incubated in a Petri dish for 2 h at 30°C (germinated kiwi pollen, GKP); alternatively, pollen was hydrated in the humid chamber, under the same conditions and for the same time of GKP, and it was eventually resuspended in particle-free PBS at concentrations of 0.5 mg/mL (hydrated kiwi pollen, HKP). For FM4-64 staining, a third treatment group was added (PBS-hydrated kiwi pollen, PKP), by resuspending the rehydrated pollen in particle-free PBS (Dubecco’s PBS 1x, Capricorn Scientific, Italy) at concentrations of 1 mg/mL, and incubating it in a Petri dish for 2 h at 30°C. PKP was used as control only in the FM4-64 experiment, since the extended incubation in a liquid medium without the promotion of germination represents a stressful condition for pollen grains (Siriwattanakul et al., 2019; Božič and Šiber, 2022), hence cannot be assumed as a neutral treatment for a negative control. For all groups, viability and germinability were estimated in bright field microscopy with a Leica DM750 microscope, equipped with a Leica ICC50 W camera, using Leica AirLab software. Only pollen that had a viability rate over 80%, a germination rate over 60% for GKP, and a germination rate of 0% for HKP and PKP was used for subsequent analyses.

### 2.3 Extracellular vesicles isolation

EVs isolation was carried out as previously described (Prado et al., 2014; Prado et al., 2015; Furini et al., 2018), with minor modifications. Briefly, pollen grains were pelleted at 5000 × g for 15 min at 4°C. Pelleted pollen was stored at 4°C until protein extraction, while the supernatant was filtered twice in 0.22 µm syringe filters, and then EVs were pelleted by ultra-centrifuge at 100000 × g at 4°C for 1h. The resulting vesicle-free supernatant (EVF) was stored at 4°C until further analysis.

### 2.4 Protein isolation and quantification

Total proteins were isolated from whole pollen grains as shown in literature (Mandrone et al., 2019), with minor modifications. Briefly, pelleted pollen was resuspended in pollen extraction buffer (PEB) (Tris-HCl 20 mM pH 8.5, DTT 2 mM, protease inhibitors cocktail 1:100) and pottered 80 times. Pollen wall debris were discarded afterwards by spinning the samples at 1000 × g for 10 min, and the supernatant (total lysate, TL) was then collected.

Total proteins were extracted from the pelleted EVs by resuspending them in PEB.

Proteins in the EVF were precipitated with 10% TCA and washed with acetone at -20°C, pelleted by centrifuge at 15000 × g for 5 min at 4°C, and then resuspended in PEB (Furini et al., 2018).

Protein content was quantified by Bradford assay (Bradford Reagent, Sigma-Aldrich, Italy).

### 2.5 Immunoblotting

Total proteins from TLs, EVs, and EVFs fractions of both treatment and control groups were resolved in one-dimensional SDS-PAGE and blotted onto nitrocellulose membrane. For each pollen sample (40 mg), the total protein content for EVs and EVFs fractions was loaded into the gel for both treatments. Instead, for TLs only 50 µg of total proteins were used, to avoid overloading. The membrane was incubated for 10 min with Ponceau staining to visualize protein profiles and loadings for all the fractions. The membrane was then blocked in 5% Blotting Grade Blocker (BioRad, Italy) in TBS for 30 min, and thus incubated at 4°C overnight with one of the following rabbit polyclonal antibodies: 1:2000 dilution of anti-clathrin heavy chain (Agrisera), 1:5000 dilution of anti-H+ATPase (Agrisera, Italy), 1:500 dilution of anti-COXII (Agrisera, Italy), 1:5000 dilution of anti-UGPase (Agrisera, Italy), 1:1000 dilution of anti-ARF1 (Agrisera, Italy), or 1:1000 dilution of anti-ALIX (Covalab, Italy). All membranes were then washed in TBS-Tween (0.05% v/v) and TBS, and they were incubated at room temperature for 2 h with 1:5000 goat polyclonal anti-rabbit IgG peroxidase conjugated (Sigma-Aldrich, Italy). Finally, the membranes were developed with Amersham™ ECL Prime Western Blotting Reagents (GE Healthcare, Italy) and read in chemiluminescence with Azure 280 (Azure Biosystems, California). Experiments were repeated in triplicate for each target protein. Comparison between proteins bands was performed using ImageJ (Schneider et al., 2012).

The existence of plant homologs for human ALIX in Actinidia chinensis Planch. was assessed using BLAST (Altschul et al., 1990; Boratyn et al., 2012) to compare human ALIX sequence to published protein sequences in Actinidia chinensis var chinensis.

### 2.6 Proteomics

EVs from 3 germinated samples were lysed in RIPA buffer (25 mM TrisHCl pH 7.2, 150 mM NaCl, 2 mM EDTA, 5% (w/v) Sodium deoxycholate, 1% (v/v) NP40, 0.1% (w/v) SDS), and their protein content was quantified by Bradford assay, BCA assay, and 2-D Quant Kit (GE Healthcare, Italy), resulting in an average of 40 μg per sample. Proteins were lyophilized and stored at -80°C until analysis. EVs protein lysates were processed (reduced and alkyklated) and trypsinized using S-trap micro methodology (Protifi, USA). Samples were resuspended to 1 μg/μL in 5% acetonitrile (v/v) and 0.1% formic acid (v/v) in a two-stage process.

Individual samples and a pool of all three samples were analysed by RP-HPLC-ESI-MS/MS using a TripleTOF 6600+ mass spectrometer (MS) in data dependent acquisition mode (Top 30), according to literature (Furini et al., 2018) with some modifications. RP-HPLC mobile phases were solvent A (0.1% (v/v) formic acid in LC/MS grade water) and solvent B (LC/MS grade acetonitrile containing 0.1% (v/v) formic acid). Samples were injected (trap/elute via 5 x 0.3 μm YMC Triart C18 trap column) onto a YMC Triart-C18 column (15 cm, 3 μm, 300-um ID) at 5 μL/min using a microflow LC system (Eksigent ekspert nano LC 425) with an increasing linear gradient of B going from 3% to 30% in 68 min; to 40% at 73 min then washing to 80% for 3 min before re-equilibration in a total time of 87 min. Datafiles were searched both individually and together using ProteinPilot 5.0.3 (Sciex) against the Actinidia chinensis Swissprot database with iodoacetamide selected as the alkylation method with the parameters of ID focus: Biological modifications and thorough ID. Protein families, cellular localizations and functions were drawn from UniProtKB (The UniProt Consortium, 2021) and from literature (Kawai and Uchimiya, 1995; Anderson et al., 2004; Tiwari et al., 2005; Al-Whaibi, 2011; Olvera-Carrillo et al., 2011; Lu et al., 2012; Suhandono et al., 2014; Dumont et al., 2016; Kuttiyatveetil and Sanders, 2017; Saqib et al., 2019). Gene ontology (GO) analysis was performed using the overrepresentation test of Panther Classification System (Thomas et al., 2022) version 17.0, with Fisher’s exact test and False Discovery Rate correction. GO terms were clustered and summarized using REVIGO with simRel as semantic similarity measure (Supek et al., 2011). Protein-protein networks were elaborated in STRING version 11.5 (Szklarczyk et al., 2021).

### 2.7 Nanoparticle tracking analysis

Nanoparticle tracking analysis (NTA) was performed using ZetaView® Basic-NTA (Particle Metrix, Germany). Pelleted EVs were resuspended in particle-free PBS and analyzed in measurement mode “Size Distribution”, 2 Cycles, 11 Positions. Germination medium and particle-free PBS were used as blank for the treatment and the control group respectively, and the measurements were performed in triplicate for each group. Eventually, particle concentrations of the blank controls were subtracted from the particle concentrations of the samples.

### 2.8 Atomic force microscopy

Atomic force microscopy (AFM) was performed using a Dimension ICON atomic force microscope equipped with a Nanoscope V controller operating in ScanAsyst tapping mode air environment. Standard silicon nitride triangular cantilevers (ScanAsyst-air, Bruker, U.K.), with resonant frequencies ranging between 45 and 95 kHz, and spring constants ranging between 0.2 and 0.8 N/m, were used. Imaging was performed at a rate of 0.7 Hz. All measurements were performed in temperature (23°C ± 1°C) controlled laboratories.

EVs fractions from GKP and HKP were resuspended in particle-free PBS at concentrations of 10.4 ·104 particles/μL (calculated using ZetaView™) for both treatment and control group. Particle-free PBS and germination medium were used as blank control to rule out contamination, following the same protocol used for EVs samples. Slides were then prepared under laminar flow hood according to literature (Sebaihi et al., 2017), with some modifications. 35 μL of sample were added on a 5 cm^2^ polylysine-coated MICA. After 1 h of incubation the samples were gently rinsed with ultrapure water and let dry overnight at room temperature. 2 x 2 μm^2^, 10 × 10 μm^2^, and 500 x 500 nm^2^ AFM images were recorded at 512 sample lines. All the images were analyzed using Nanoscope software. A first order flattening was applied to AFM images and the section analysis function of Nanoscope software was used to detect and measure the size of the particles.

### 2.9 Fluorescence microscopy

Fluorescence microscopy was performed using a NEXCOPE NE920 microscope equipped with a mercury short-arc lamp Osram HBO 103 and a 20 Mpx cooled color camera with C-MOS 1” sensor (TiEsseLab, Italy).

To prove the vesicular nature of the isolated nanoparticles, both whole germinated pollen grains and the EVs fractions from GKP, PKP, and HKP were resuspended in PBS added with 2 μM FM™ 4-64 (also known as SynaptoRed™ C2) fluorescent dye (Tocris, Italy) and observed at the fluorescence microscope using TRIC filter. Intact pollen grains hydrated in humid chamber or in PBS were not considered for this analysis, since the pollen wall would not allow the visualization of the fluorescent signal inside the cytoplasm.

For fluorescence immunolocalization of ALIX-homologs, only germinated pollen samples were analyzed. Germination medium was discarded after a light centrifugation (1000 × g), and pelleted pollen grains were then processed as described in literature (Parrotta et al., 2018; Mandrone et al., 2019), with some modifications. Briefly, pelleted pollen grains were fixed with PME buffer (4% formaldehyde, 50 mM PIPES pH 6.8, 1 mM MgSO_4_, 5 mM EDTA) for 1 h, digested by pectinase and cellulase for 7 min, permeabilized with 0.5% Triton X-100 for 30 min, and eventually dehydrated in cold methanol (-20°C) for 10 min. Samples were blocked with 3% BSA in PBS, and then incubated with a 1:50 dilution of anti-ALIX in PBS at 4°C overnight. Samples used as negative controls were incubated with PBS only. All samples were thus incubated with a goat polyclonal anti-rabbit IgG, FITC-conjugated (dilution 1:200, SouthernBiotech, Italy), and 3% BSA in PBS, for 2 h at room temperature, in the dark. Finally, samples were washed in PBS, added with 10% glycerol, and mounted on glass slides. Fluorescence was observed at 600X magnification using the FITC filter.

### 2.10 Electron microscopy

For electron microscopy, the transmission electron microscope (TEM) Philips Morgagni 268 D set at 80 kV was employed, and images were captured with a MegaView II CCD Camera (Philips Electronics, The Netherlands) and analyzed with the microscope software (AnaliSYS). Immunogold labeling was carried out following literature (Parrotta et al., 2019). Briefly, germinated pollen was dehydrated in growing concentrations of ethanol, and then infiltrated with LR white resin (Sigma-Aldrich, Italy). Thus, the resin was encapsulated and polymerized in an oven at 40°C for 2 days. The resin was then sectioned, and the sections were blocked in 5% normal goat serum (Invitrogen, Italy) for 20 min and then incubated in a 1:50 dilution of the anti-ALIX antibody for 1 h. Three sections were selected as negative controls and they were not incubated with the primary antibody. Finally, the excess of primary antibody was washed in 50 mM Tris–HCl pH 7.6, 0.9% NaCl, 0.1% Tween, and all the sections were incubated for 45 min with a dilution 1:20 of goat anti-rabbit secondary antibody conjugated with 15 nm gold particles (BioCell, Italy). Sections were washed with distilled water and counterstained first with 2% uranyl acetate for 10 min, and then with lead citrate for 5 min. No osmium was employed in the preparation of the samples. At least 50 pollen tubes and grains were analyzed per sample.

### 2.11 Statistical analysis

Statistical analysis was carried out in RStudio (RStudio Team, 2020). To test datasets for normality, Shapiro-Wilk test was applied, using the “shapiro.test” function. To evaluate the statistical significance (p-value <0.05) of the differences between GKP and HKP samples, t-test and two-way ANOVA were performed on protein concentrations, using “t.test”, “lm”, and “anova” functions with default settings, followed by a post-hoc pairwise t-test with Bonferroni correction using “pairwise.t.test” function.

## 3 Results

### 3.1 Visualization and measurement of EVs

**Figure 1:**
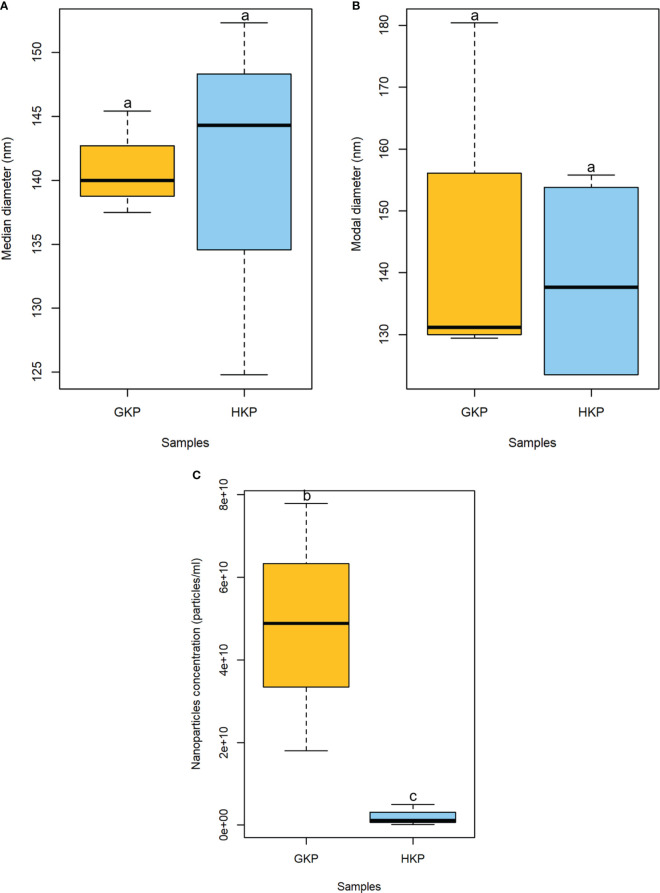
Boxplot representation (± SD) of median **(A)** and modal **(B)** diameters (nm), and concentration (particle/mL) **(C)** of nanoparticles in EVs fractions from GKP and HKP. Samples with different superscript letters differed with a weak significance (p-value<0.1) by one-way ANOVA.

Nanoparticles ranging between 15 and 266 nm in diameter were present in the EVs fractions of both GKP and HKP when assessed by NTA. However, the majority of particles (>95%) had a diameter in the range between 120 and 209 nm. While median and modal diameters did not vary considerably between the two groups, nanoparticles in GKP EVs fraction showed a trend increase in concentration than those isolated from HKP (**Figure 1**).

**Figure 2:**
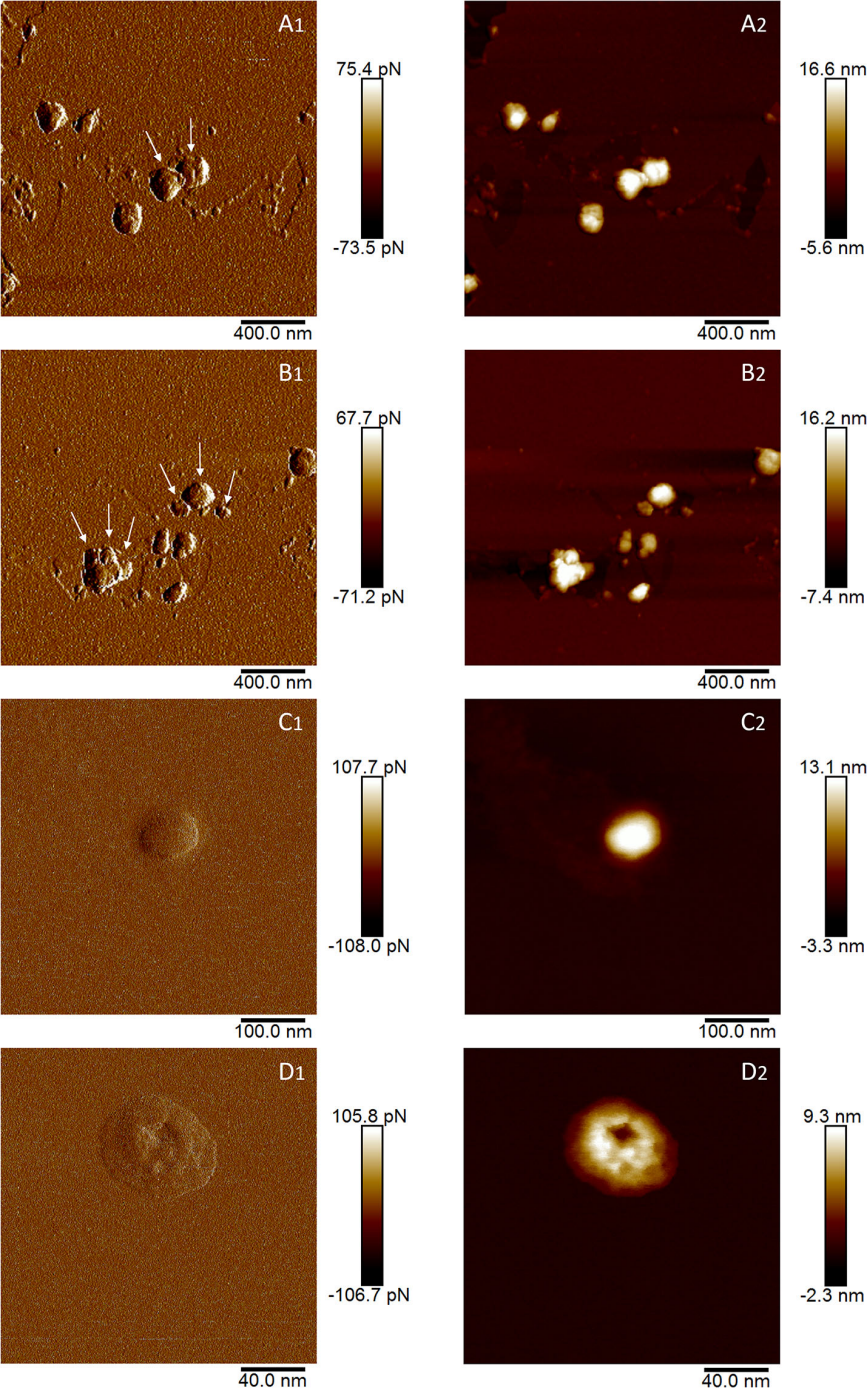
AFM images of EVs isolated from germinated **(A, B)** and hydrated **(C, D)** kiwi pollen. Images indexed with 1 (A1, B1, C1, D1) are captured in height mode, while images indexed with 2 (A2, B2, C2, D2) are captured in peak force error. In A1 and B1, arrows indicate plausible individual vesicles in vesicle aggregates.

In AFM analysis, nanoparticles compatible with EVs were visible in both GKP and HKP EVs fractions, while in the blank controls only smaller particles were present, with dimensions well below the range of published EVs (**Supplementary Figure 1**). Many particles appeared to be aggregated (**Figure 2**), having diameter and height of respectively 40.4 nm ± 60.4 nm and 10.7 ± 2.2 nm for HKP, and of 78.1 nm ± 57.4 nm and 9.8 ± 3.7 nm for GKP.

**Figure 3:**
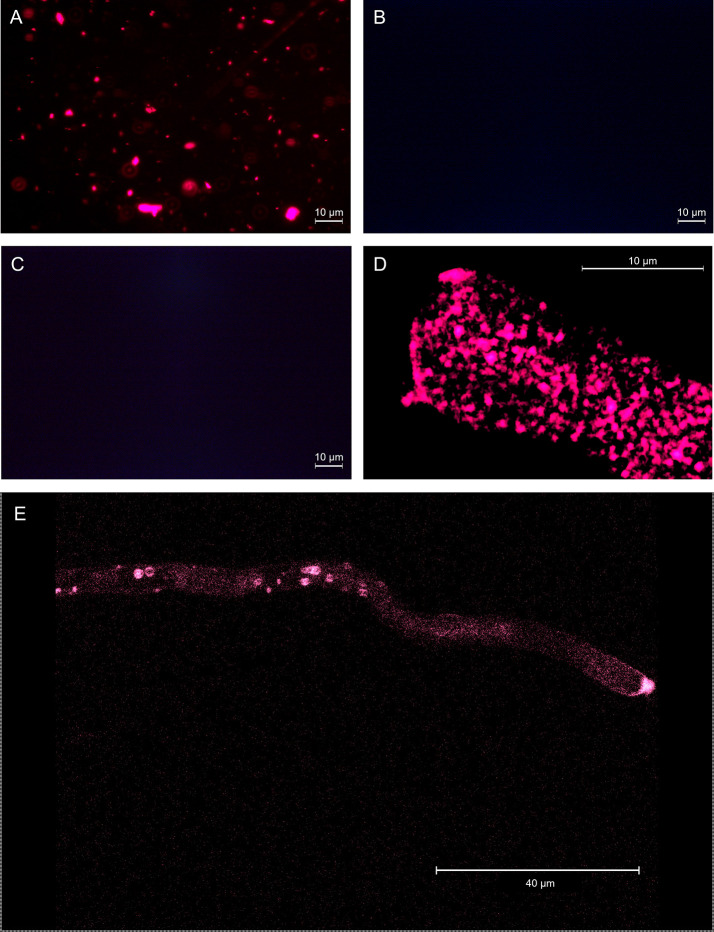
EVs from germinated kiwi pollen **(A)**, EVs from kiwi pollen hydrated in a humid chamber **(B)**, and EVs from kiwi pollen hydrated in PBS **(C)**, dyed with FM4-64™ to enhance the presence of double-layered vesicles. **(D, E)** show FM4-64™ staining on the pollen tube apical region at different magnifications, as a positive control.

GKP EVs emitted a clear fluorescent signal after FM4-64™ staining (**Figure 3A**), indicating the presence of vesicles with double-layered lipidic membranes in this fraction. Excessive dimensions of the fluorescent spots compared to the maximum expected (266 nm) was likely due to aggregation of the vesicles (as also shown by AFM) and to the diffusion of fluorescent light. In fact, while the aggregation itself does not affect the FM4-64™ staining, the presence of closely associated vesicles marked with fluorescence and observed at the optical microscope can produce larger fluorescent spots than those expected on the basis of NTA and AFM analyses, and of the filtrations employed in the isolation protocol. On the contrary, EVs fractions from HKP and PKP did not show any fluorescence after FM4-64™ dyeing (**Figures 3B, C**), along with the blank controls (**Supplementary Figure 2**). Intact germinated pollen grains were stained with FM4-64™ as a positive control (**Figures 3D, E**), showing uniform fluorescent spotting along the pollen tube (**Figure 3E**), and the characteristic intense coloration on the tube apex (**Figure 3D**), due to vesicle accumulation (Parton et al., 2003).

### 3.2 Characterization of the proteins isolated from EVs

**Figure 4:**
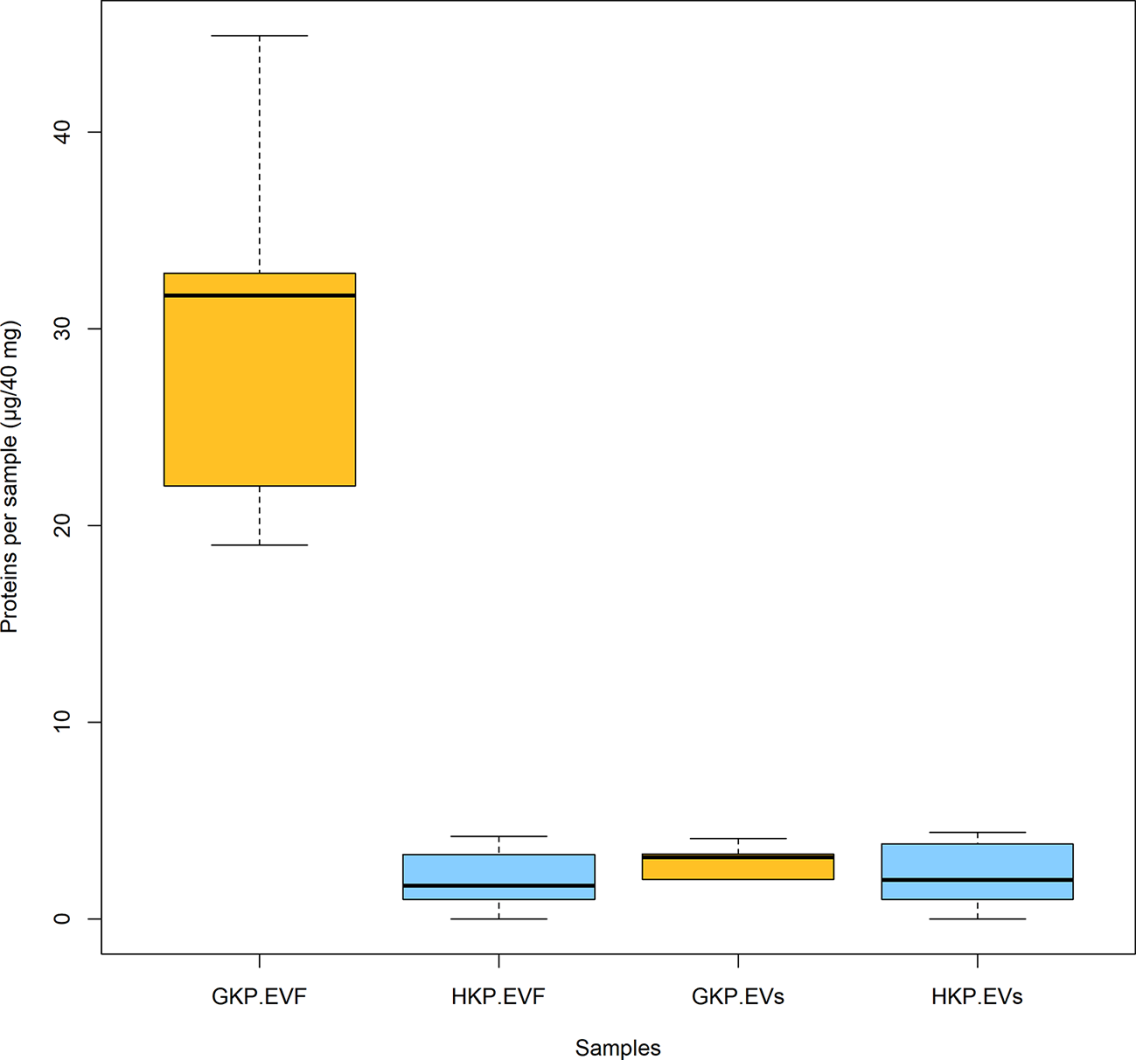
Mean protein content (± SD) for EVs and EVF from germinated and hydrated samples. Samples with different superscript letters were significantly different (p-value<0.05) by two-way ANOVA and Student t-test.

Total protein concentration of GKP EVs fraction resulted significantly higher (p-value<0.02) than the ones of HKP EVs fraction and EVFs fractions from both groups; while protein concentration of GKP EVFs, GHP EVFs, and GHP EVs fractions was comparable (**Figure 4**).

**Figure 5:**
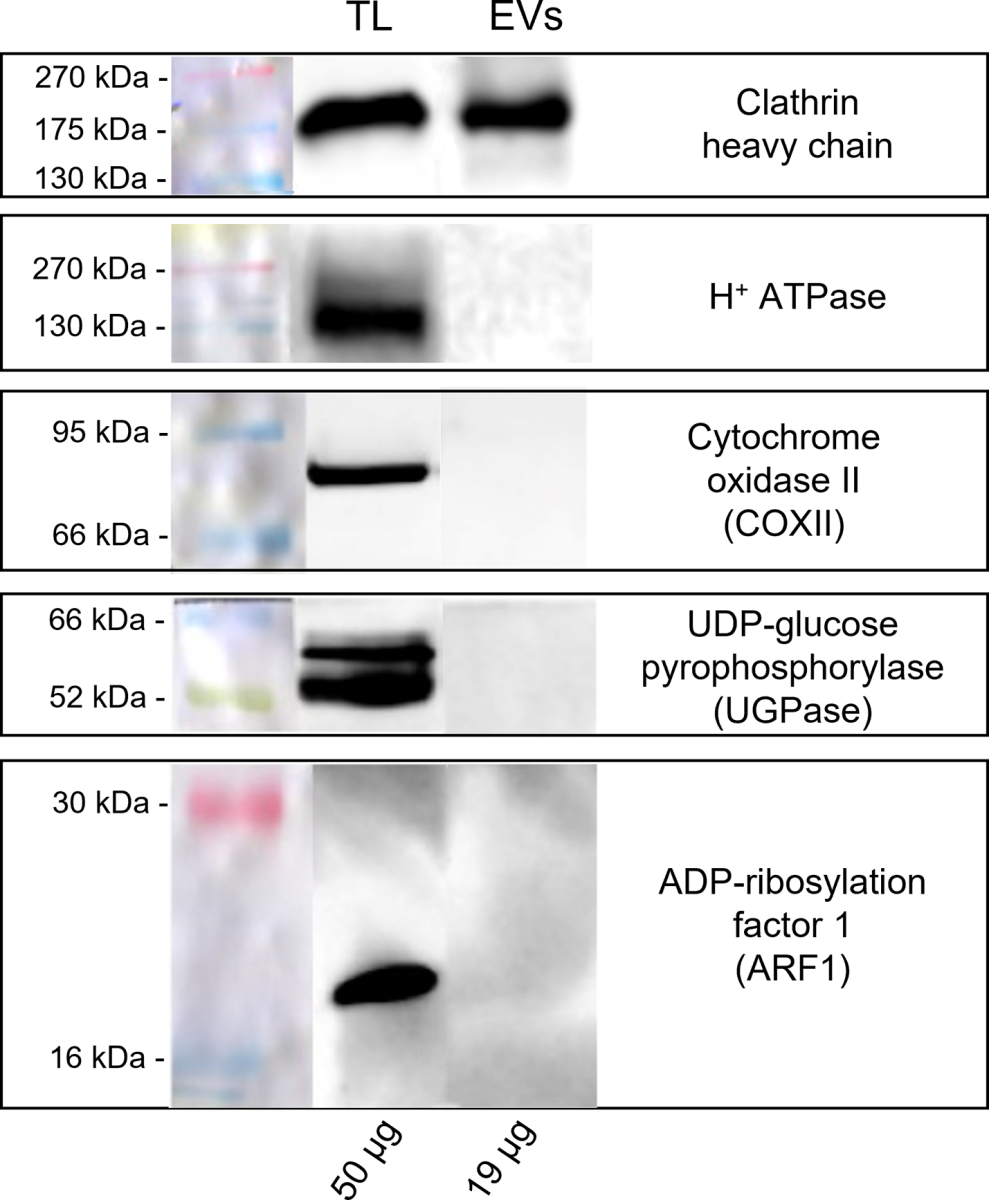
Western blot analysis of pollen total lysate (TL) and nanovesicles (EVs) fractions from germinated kiwi pollen (GKP). Samples were probed for the presence of molecular markers of different cell compartments with the following rabbit polyclonal antibodies: 1:2000 dilution of anti-clathrin heavy chain, 1:5000 dilution of anti-H+ATPase, 1:500 dilution of anti-COXII, 1:5000 dilution of anti-UGPase, 1:1000 dilution of anti-ARF1. The membranes were developed with ECL and read in chemiluminescence. On the left are reported the molecular weights of the pre-stained ladder, while on the bottom are reported the protein amounts loaded per lane.

A Western blot analysis was carried out to identify the subcellular origin of the pollensomes and to rule out the presence of contaminations due to possible pollen tube ruptures during EVs isolation. Ponceau staining (**Supplementary Figure 3**) revealed different protein profiles for all three fractions of GKP, with distinct bands in the EVs fraction between 60 and 45 kDa, and between 35 and 25 kDa. Since protein quantification and Ponceau staining (**Supplementary Figure 3**) indicated a negligible protein content in both EVF fractions and in HKP EVs fraction, only TL and EVs from GKP were analyzed by immunoblotting. Clathrin heavy chain was chosen as a marker for vesicular compartments, H+ATPase as plasma membrane marker, COXII (Plant Cytochrome oxidase subunit II) as mitochondrial marker, UGPase (UDP-glucose phosphorylase) as cytoplasmic marker, and ARF1 (ADP-ribosylation factor 1) as a marker for the Golgi membrane. All the markers were found in GKP TL, which was expected, but only clathrin heavy chain was present in GKP EVs fraction (**Figure 5**).

**Figure 6:**
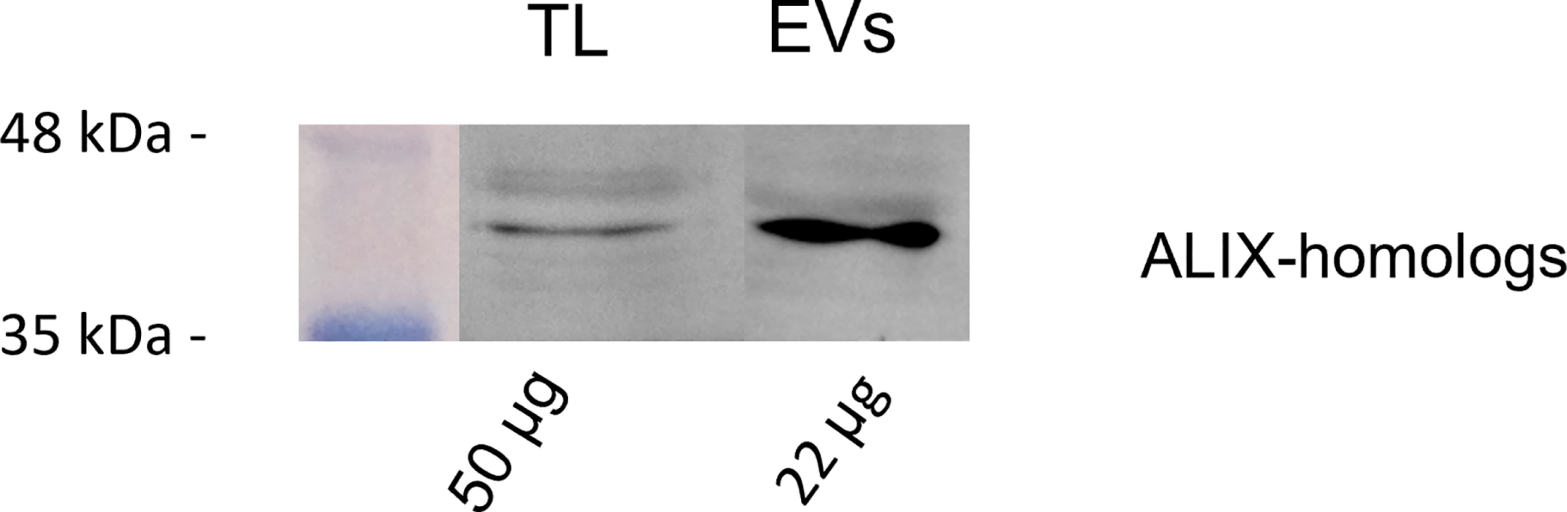
Western blot analysis of pollen total lysate (TL) and nanovesicles (EVs) fractions from germinated kiwi pollen (GKP), probed with a 1:1000 dilution of anti-ALIX rabbit polyclonal. The membranes were developed with ECL and read in chemiluminescence. On the left are reported the molecular weights of the pre-stained ladder, while on the bottom are reported the protein amounts loaded per lane.

A preliminary BLAST analysis revealed the presence of a Bro-1 domain containing protein (encoded by the gene CEY00_Acc28537) in the genome of Actinidia chinensis Planch., which showed 93.78% sequence identity with human ALIX. Western blot analysis using anti-human ALIX polyclonal antibodies revealed the presence of a possible plant homolog of ALIX, with a molecular weight around 43 kDa, in both TLs and in GKP EVs. Moreover, this protein showed a four to fivefold band intensity in the KPG EVs fraction when compared to the TLs, even though the protein content of the former was half the one of the latter (**Figure 6**).

The RP-HPLC-ESI-MS/MS provided a list of 2903 accession numbers matching with the peptides sequenced from GKP EVs, corresponding to 1170 different protein IDs (**Supplementary Table 1**). Among these, there were two tetraspanins (tetraspanin-8 like and tetraspanin-15 like), which are multi-pass membrane proteins, confirming the presence of membrane-bound particles in the GKP EVs fraction. ESCRT-related proteins, which have membrane-binding activity and allow the EVs to fuse with membranes, and heat-shock proteins (Hsp70 and Hsp90), which are cytosolic proteins commonly incorporated in EVs, were also present (**Supplementary Table 1**).

**Figure 7:**
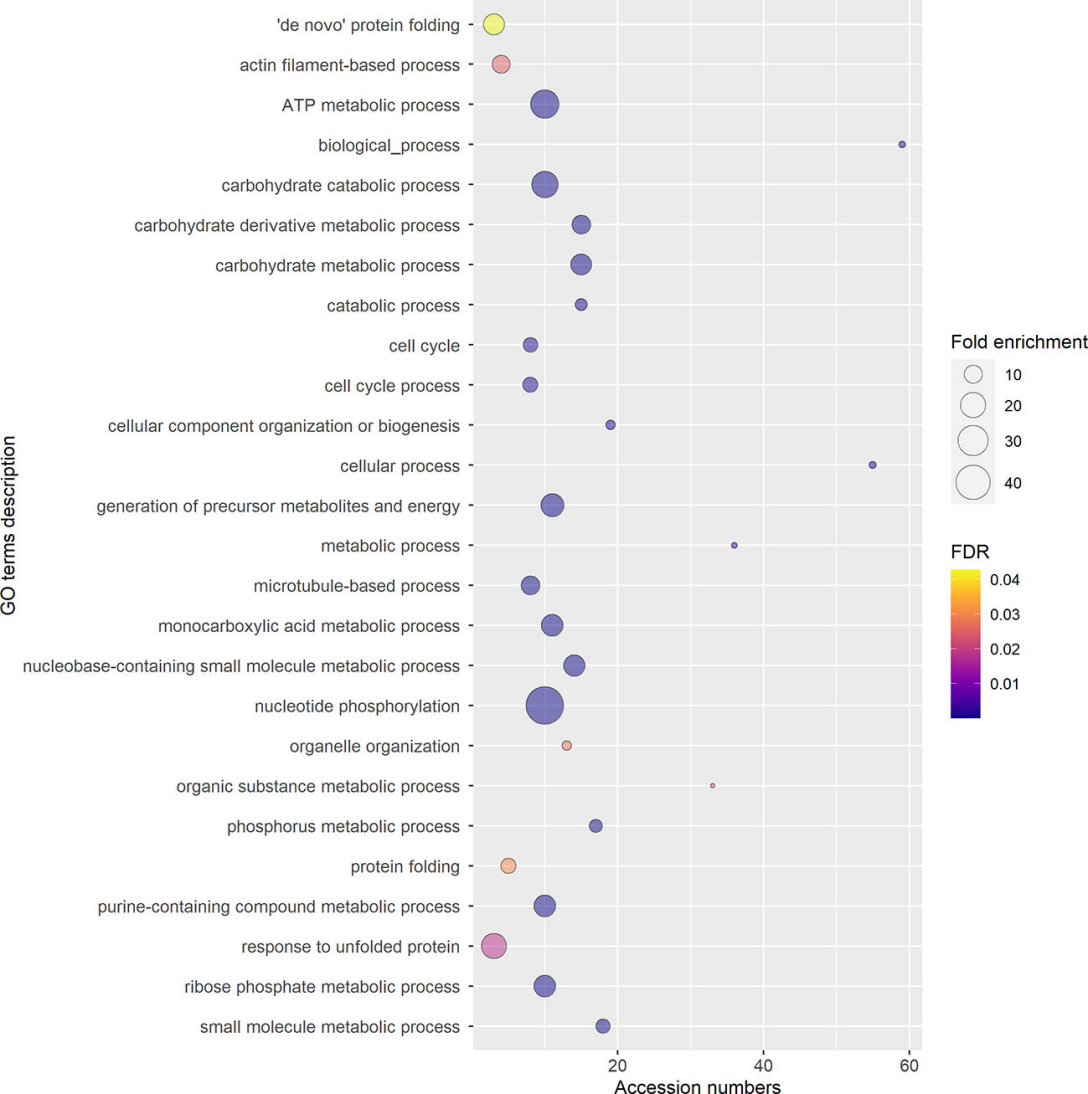
Gene Ontology (GO) terms for Biological Process, relative to the 97 selected accession numbers identified in extracellular nanovesicles from germinated pollen of *Actinidia chinensis* Planch. GO terms were provided by Panther Classification System version 17.0 and shortened by REVIGO using SimRel similarity measure. The diameter of the bubbles indicates the overrepresentation of the term when compared to the whole Actinidia chinensis Planch. genome (Fold enrichment), while the color indicates the False Detection Rate (FDR) value. GO terms with FDR>0.05 are not shown.

**Figure 8:**
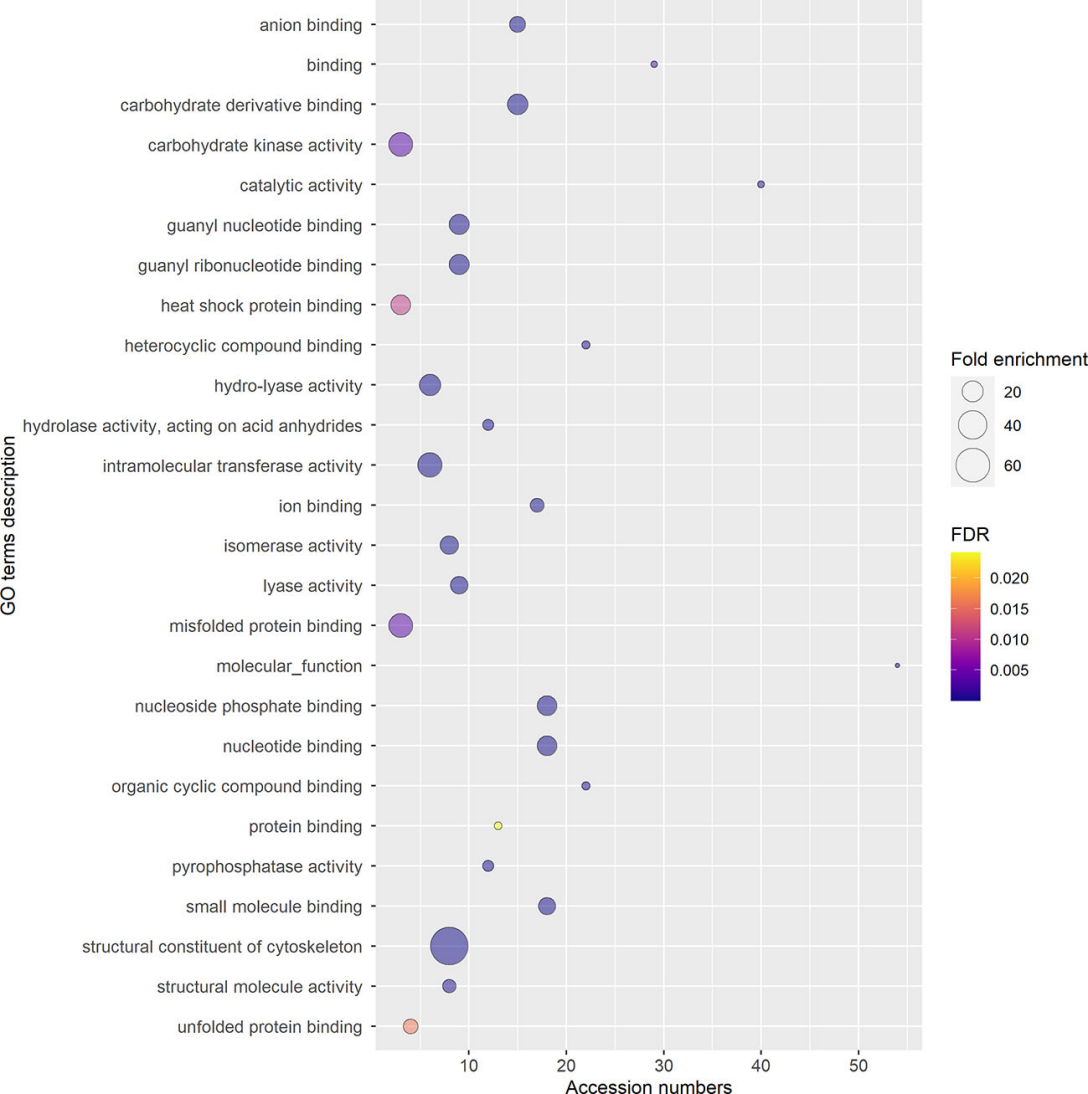
Gene Ontology (GO) terms for Molecular Functions, relative to the 97 selected accession numbers identified in extracellular nanovesicles from germinated pollen of *Actinidia chinensis* Planch. GO terms were provided by Panther Classification System version 17.0 and shortened by REVIGO using SimRel similarity measure. The diameter of the bubbles indicates the overrepresentation of the term when compared to the whole Actinidia chinensis Planch. genome (Fold enrichment), while the color indicates the False Detection Rate (FDR) value. GO terms with FDR>0.05 are not shown.

To ensure a better accuracy of the protein identification, only the accession numbers present in all three replicas, identified using at least 2 peptides, and with a 95% confidence coverage over 70% of the peptide length were selected for GO analysis, obtaining a list of 97 accession numbers, corresponding to 51 different protein IDs (**Supplementary Table 2**). In fact, some of the proteins listed in **Supplementary Table 1** are unlikely to be secreted (e.g. ATP-synthase subunit beta, enzymes from the TCA cycle, nuclear proteins), and represent possible misidentifications. According to STRING, the 97 proteins selected had significantly more interactions (p-value<0.01) than a comparable random group of proteins drawn from kiwi genome, implying their biological connection and possible common role (**Supplementary Figure 4**). The majority of the accession numbers examined were involved in biological processes (BP) related to cellular component organization or biogenesis, and in metabolic processes of phosphorus and carbohydrates (**Figure 7**; **Supplementary Figure 5**), which is compatible with the high metabolic activity in the pollen cytoplasm during pollen tube elongation. Panther results indicated that, out of 78 GO terms in the BP category, 77 were overrepresented when compared to Actinidia chinensis proteome in UniProt database, with nucleoside metabolic processes and glycolytic processes having the highest fold enrichment (**Figure 7**; **Supplementary Figure 5**). Three proteins among those analyzed are also involved in stress response: the late embryogenesis abundant protein, the stress-induced protein, and the Hsp70. Others are involved in transport and signaling, like the guanosine nucleotide diphosphate dissociation inhibitor, and the clathrin heavy chain like protein (The UniProt Consortium, 2021). Furthermore, this analysis revealed the presence in GKP EVs of Ole e 1, a major pollen allergen with the biological function of trypsin inhibitor, also regarded as a pollensome marker (Prado et al., 2014; Prado et al., 2015). The most frequent Molecular Functions (MF) among the analyzed accession numbers were catalytic and binding activities, in line with the BP results (**Figure 8**; **Supplementary Figure 6**). While all the identified GO were overrepresented in the sample, “fructose-bisphosphate aldolase activity” and “structural constituent of cytoskeleton” were the GO terms with the highest fold enrichment (>75). Proteins involved in the cell wall remodeling, like α-L-arabinofuranosidase, glucosidases, galactosidases, and pectinases have been identified as well (**Supplementary Tables 1**, **2**). Concerning the subcellular origin of the analyzed proteins, the majority of them belonged to the cytoplasm or to a protein-containing complex, while the most overrepresented GO terms for Cellular Components (CC) were the “lid subcomplex of the proteasome regulatory particle” and the “proteasome complex” (**Supplementary Figure 7**).

### 3.3 Localization of ALIX-homologs in the pollen tube

**Figure 9:**
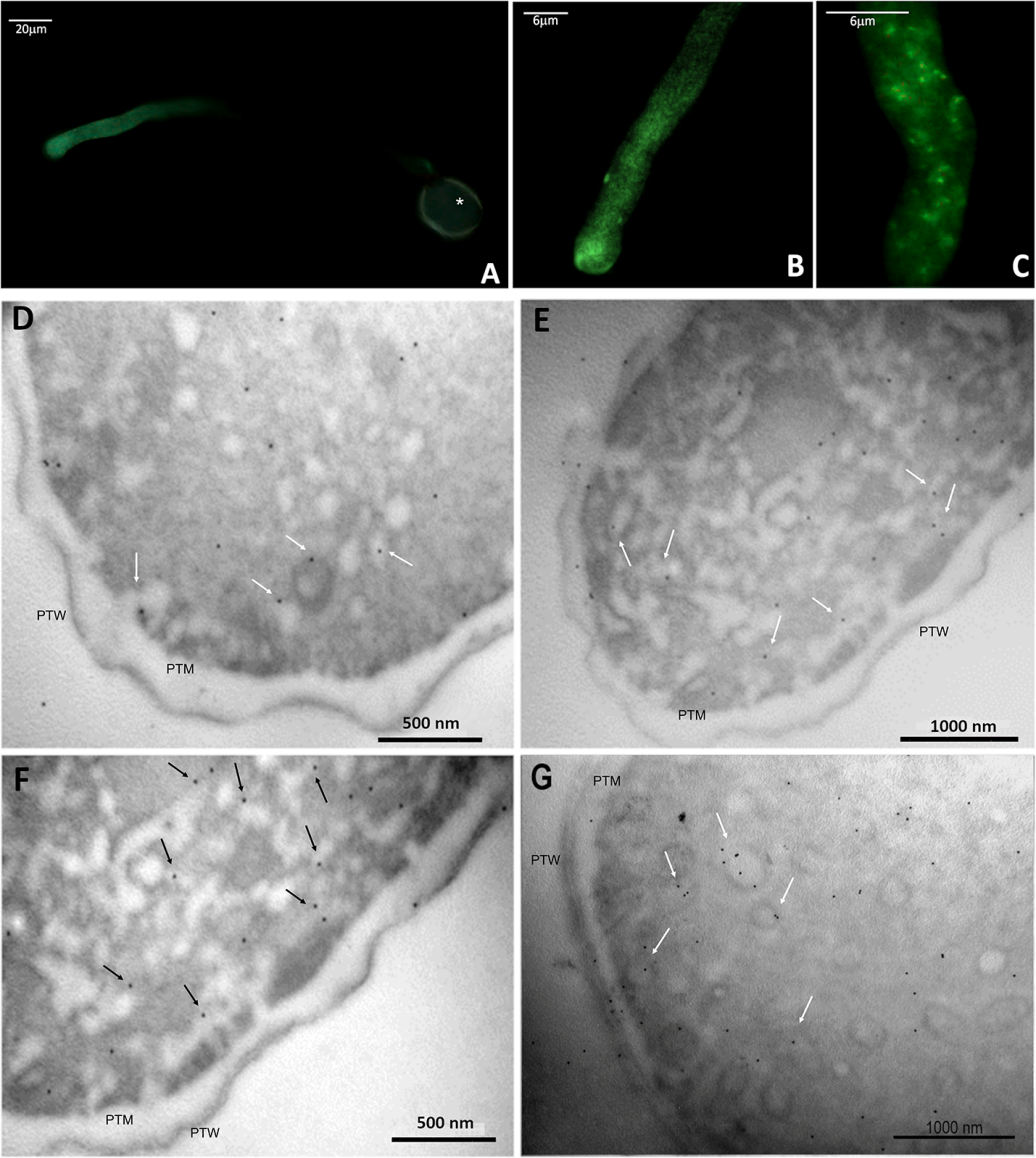
Immunolocalization of ALIX-homologs in kiwi pollen tube. **(A–C)**: indirect immunofluorescence labeling with FITC-conjugated secondary antibodies, probed for ALIX-homologs, at different magnifications.Tthe fluorescent labeling of the target proteins is pictured in green. In A, the autofluorescence of the pollen wall is visible (*). **(D–F)**: immunogold labeling of ALIX-homologs in transverse sections of kiwi pollen tube. Images were taken at the level of apex and subapex. Some of the proteins appear associated with subcellular structures compatible with vesicles (arrows), near the plasma membrane. PTW, pollen tube wall; PTM, pollen tube plasma membrane.

Immunofluorescence labeling revealed the presence of ALIX-homologs in the pollen tube, localized almost homogeneously in its metabolically active portions (**Figures 9A–C**), with a slight increase towards the apical region (**Figures 9A, B**). The negative control excluded nonspecific fluorescence for the pollen tube (**Supplementary Figure 8**).

The distribution of ALIX homologues was also studied by immunogold electron microscopy. **Figures 9D–G** show that these proteins were distributed in two different ways: in the cytoplasm of pollen tubes, and in association with rounded/tubular membrane-bounded structures. Since the images in **Figure 9** were collected primarily at the apex and subapex of pollen tubes, it is likely that the membrane structures correspond to secretory or recycling vesicles, but other intermediate compartments cannot be excluded. In some cases, the signal was also found in association with the plasma membrane, although it was not a consistent trait, while it was never found in association with the cell wall. The negative control (**Supplementary Figure 8**) confirmed that nonspecific binding of gold-conjugated secondary antibodies did not impair the analysis.

## 4 Discussion

### 4.1 Nanoparticles can be isolated from kiwi pollen by ultracentrifugation

This study demonstrated that nanoparticles can be isolated from kiwi pollen samples after ultracentrifugation at 100000 × g (**Figures 1**, **2**). While this has already been established for germinated pollen of other species (Prado et al., 2014; Prado et al., 2015), it is to our knowledge the first time that such nanoparticles have been also isolated from hydrated pollen, although Grote and colleagues described similar nanoparticles released after hydration in rainwater by birch, alder, hazel, and ryegrass pollen, using electron microscopy (Grote et al., 2000; Grote et al., 2003). However, while pollen types studied by Grote and colleagues underwent germinative abortion during hydration, kiwi pollen used in this study showed 0% germination rate after hydration, performed either in humid chamber or in PBS. It is clear that hydrated kiwi pollen releases a considerably smaller amount of nanoparticles than germinated kiwi pollen (**Figure 1**). When samples containing the same concentration of nanoparticles isolated from HKP and GKP were visualized in AFM, it was possible to appreciate rounded, vesicle-like structures (**Figure 2**) in both groups, compatible for dimensions and shape with small EVs (Prado et al., 2014; Prado et al., 2015; Kurian et al., 2021).

### 4.2 Nanoparticles released by germinated kiwi pollen are EVs

When the nanoparticles (EVs fraction) isolated from the same sample size of GKP, HKP, and PKP were stained with FM4-64™, only the GKP preparation showed a detectable fluorescent signal, meaning that the nanoparticles isolated were EVs with a double-layered lipidic membrane (**Figures 3A, B**). The absence of a fluorescent signal in PKP nanoparticles indicates that the secretion of such vesicles is not promoted by the resuspension of pollen in a liquid medium, but rather by the germination process itself (**Figure 3C**). The relatively high protein content of GKP EVs fraction (**Figure 4**) corroborates the idea that GKP secretes nanovesicles, and the low protein concentrations in EVF fractions seem to exclude the possibility of a significant contamination of the EVs fraction by secreted proteins. Although in the light of NTA and AFM results (**Figures 1**, **2**) it is not possible to rule out that HKP can secrete EVs, it is apparent that, if present, its secretion rate is very low and almost undetectable. Hence, while kiwi pollen hydration is necessary to reactivate pollen metabolism and promote the pollen tube growth, the hydration itself does not seem to activate any secretion pathway unless it is associated with stigmatic stimuli. This is in line with the current theory that pollen-pistil interactions during hydration are driven by proteins already present on the pollen surface, and specifically in the pollen coat (Wang et al., 2017). Cases in which the secretion of EVs-like nanoparticles has been documented as a consequence of pollen rehydration (Grote et al., 2000; Grote et al., 2003) always involved species in which pollen germination can be triggered by the resuspension in a liquid medium, while the present paper suggests that pollen rehydration caused by high relative humidity cannot trigger any relevant EVs secretion.

To furtherly assess that proteins and membranes found in GKP EVs fraction did not derive from pollen tube rupture and cell debris, total protein content of TL and EVs fractions from GKP samples was probed for known molecular markers of different organelles. The immunoblotting analysis excluded the presence of contaminations from plasma membrane, mitochondria, cytoplasm, and Golgi apparatus in EVs fractions (**Figure 5**), confirming that pollen integrity was preserved throughout the EVs isolation process. Moreover, proteomic analysis assessed the presence of tetraspanins, ESCRT-related proteins, and Hsp (**Supplementary Table 1**) fulfilling the technical requirement of the presence of specific transmembrane and cytosolic proteins to correctly identify EVs (Théry et al., 2018). The presence in all three replicas of Ole e 1 (**Supplementary Table 1**), which was described as a pollensome molecular marker by Prado and colleagues (Prado et al., 2014; Hafidh et al., 2016), furtherly validate the presence of extracellular nanovesicles in the GKP EVs. Similarly to those found in pollensomes (Prado et al., 2014), many proteins from GKP EVs are involved in metabolic and biosynthetic processes, cell signaling, vesicular trafficking, cytoskeletal movements, and stress response (see **Supplementary Materials**). Moreover, enzymes thought to be involved in the cell wall reorganization needed for vesicles secretion (Woith et al., 2021) have been identified in GKP EVs as well (**Supplementary Table 1**).

### 4.3 EVs secreted by germinated kiwi pollen could be plant exosomes

The EVs isolation employed hereby was originally designed for mammalian exosomes (Prado et al., 2014; Furini et al., 2018; Kurian et al., 2021). The nanoparticles isolated in this study resulted on average larger in size than those described as pollensomes (Prado et al., 2014), but nonetheless their modal and median diameters fell within the accepted range for exosomes (**Figure 1**) and are compatible with published plant exosomes dimensions (Rutter and Innes, 2017). The tendency to aggregation shown by these particles during NTA, AFM, and FM4-64™ staining (**Figures 2**, **3**) and previously reported in literature (Linares et al., 2015) might explain the difference with the published pollensomes dimensions. In fact, ZetaView camera tends to consider the whole aggregate as a single particle, possibly overestimating mean and mode values of the diameter, and underestimating particle concentrations.

Most of the enzymes present in GKP EVs were involved in catabolic processes, especially in the energetic metabolism, with glycolytic activity and nucleoside metabolic activity (**Supplementary Figures 4**, **5**). Since pollen tube elongation must be sustained by an intense metabolic activity, the cytoplasmic concentrations of metabolism-related enzymes are particularly high in this phase (Wang et al., 2022), hence they are more likely to be randomly internalized during EVs formation. Moreover, some of them, such as the pectinesterases or the pollen-specific leucine-rich repeat extensin-like protein (**Supplementary Table 1**), can cooperate in the degradation of female tissues to open a passage for the pollen tube through the style (Chae and Lord, 2011), and they might be released in a membranous compartment to extend their extracellular half-life. Another important feature of these vesicles, that emerged from the GO and interaction analyses, is the presence of interconnected proteasome subunits, 26S proteasome regulatory subunits, and proteasome inhibitors and activators, which are unlikely to have been randomly sorted into the vesicles (see **Supplementary Materials**). In fact, the EVs also contained various kinases, ubiquitin, and ubiquitin-related enzymes. It is known that proteasome plays a pivotal role in regulating the phosphoproteome of kiwi pollen during the tube formation and growth, also affecting endocytosis and vesicular trafficking (Vannini et al., 2019). However, the extracellular role of the proteasome has been extensively studied only in mammals, where its presence in EVs, and especially exosome-like vesicles, appears to be related to pathological conditions, possibly targeting misfolded proteins in neighboring cells or in extracellular fluids (Ben-Nissan et al., 2022).

Ubiquitin-like proteins and ubiquitin-related enzymes are also common in both plant and mammalian exosomes, along with other proteins revealed by the proteomic analysis of GKP EVs, such as HSP70, chaperones, syntaxins, actin, and tetraspanins (**Supplementary Tables 1**, **2**) (Johnstone, 2006; Rutter and Innes, 2017; Jeppesen et al., 2019; Kurian et al., 2021; Boccia et al., 2022). Ras-related Rab proteins (**Supplementary Table 1**), which have been considered as possible markers for plant exosomes (Regente et al., 2009), were also found. Moreover, using a polyclonal anti-ALIX antibody it was possible to detect the presence of a putative ALIX-homolog in GKP EVs (**Figure 6**). This protein was clearly more concentrated in EVs than in TL, suggesting a potential role of this protein in the formation or secretion of the nanovesicles. In fact, plant homologs of ALIX are known to participate in the differentiation of MVBs and to be involved in vesicular trafficking (Kalinowska et al., 2015; Cui et al., 2016; García-León and Rubio, 2020). The protein band had a molecular weight of about 43 kDa, that is compatible with the molecular weight estimated for ALIX-homolog encoded by the gene CEY00_Acc28537 of Actinidia chinensis var. chinensis (The UniProt Consortium, 2021). The immunofluorescence labeling of ALIX-homologs allowed to visualize their homogeneous distribution in the metabolically active portion of the elongating pollen tube (**Figures 9A–C**). This was confirmed by immunogold labeling that localized the proteins both in the cytoplasm and associated with vesicle-like structures near the pollen tube membrane (**Figures 9D–G**), which is compatible with the known subcellular localization and mechanisms of ALIX and Bro1 domain-containing proteins (Bissig and Gruenberg, 2014; García-León and Rubio, 2020), and with the observed localization of Ole e 1 in the pollen tubes (Prado et al., 2014).

Immunoblotting also revealed the presence of clathrin heavy chain in GKP EVs fraction (**Figure 5**). Clathrin light and heavy chains, clathrin heavy chain like proteins, clathrin coat assembly proteins, and clathrin interactors EPSIN like were also identified in GKP EVs by RP-HPLC-ESI-MS/MS analysis (**Supplementary Tables 1**, **2**). Since clathrin mediates endocytic processes during the pollen tube elongation (Blackbourn and Jackson, 1996; Narasimhan et al., 2020), its presence in the EVs fraction may support the hypothesis of Prado and collaborators that these extracellular nanovesicles could have an endocytic origin (An et al., 2007; Prado et al., 2014). In fact, while it is not considered an exosome marker, clathrin heavy chain is often found in exosomes (Woith et al., 2021). It is possible that during the formation of exosomes, clathrin triskelions derived from the disassembly of endocytic vesicles coatings are still present in the cytoplasm near the endosome, and they might be incorporated in the ILVs lumen, as it happens for other cytoplasmic molecules (Kurian et al., 2021). In fact, dynamin and dynamin-related protein like, which are involved in the removal of clathrin coating from endocytic vesicles, were also identified in the GKP EVs proteome (**Supplementary Table 1**).

While proteomic analysis identified the presence of ARFs, ARF-GAPs, and ARF-GEFs in GKP EVs (**Supplementary Table 1**), ARF1, which is considered a molecular marker for Golgi membranes (Robinson et al., 2011), was not detected in EVs by immunoblotting (**Figure 5**). The absence of Golgi markers in GKP EVs could imply that these nanovesicles bypassed the Golgi apparatus and underwent unconventional secretion (Del Duca et al., 2013; Hafidh et al., 2016; Rabouille, 2017), which is in line with the exosome hypothesis. In fact, the proteomic analysis revealed the presence of proteins that have been found to be unconventionally secreted during pollen tube growth, such as actin, adenosine kinase (ADK), ARF/ARFGAP, Gp-dh-C domain containing proteins, HSP70, proteasome subunits, Ras, ribonucleoside-diphosphate reductase large subunit (RRM1), UDP-arabinopyranose mutase, and the translationally controlled tumor protein (NtTCTP) which is thought to be involved in pollen tube guidance and ovule targeting (**Supplementary Tables 1**, **2**). Contrarily, only few proteins known to be conventionally secreted and abundant in germinated pollen secretome were found in EVs proteome (Hafidh et al., 2016). An exocyst subunit Exo70 was identified in the GKP EVs (**Supplementary Table 1**), but their staining with FM4-64™ (**Figure 3**) indicates the presence of a lipidic bilayer, excluding the unconventional secretion by EXPOs, which produces single-layered extracellular vesicles (Wang et al., 2010; Hafidh et al., 2016). EVs proteomic analysis also identified proteins involved in signal transduction, e.g. a total of 277 kinases, and a cysteine protease family protein that could act as a ligand (Hafidh et al., 2016), and this is consistent with the role of exosomes in cell-cell communication.

However, as for Prado and colleagues (Prado et al., 2014) it was not possible in this study to visualize putative ILVs inside the vesicle-like structures associated with ALIX-homologs, and thus it was not possible to assess the presence of an MVB with the imaging techniques employed hereby. Nonetheless, the presence of ESCRT-related proteins in GKP EVs proteome reinforces the idea that at least a prominent subpopulation of the isolated pollen extracellular nanovesicles could derive from MVBs, since ESCRT known functions in plant cells are related to the ILVs formation and possibly to the MVB fusion with the plasma membrane (Gao et al., 2017).

## 5 Conclusions

Pollen-pistil interactions have always been a fascinating yet elusive topic in plant molecular biology. Studies on pollen secretome are starting to shade light on the molecules involved in pollen hydration, pollen-stigma compatibility, pollen tube entrance, and tube guidance through the style (Del Duca et al., 2010; Hafidh et al., 2014; Hafidh et al., 2016; Mandrone et al., 2019). Since intercellular communication is fundamental for a successful fertilization, it is plausible that bioactive molecules might be secreted by pollen through vesicles, to stabilize them during their journey towards their target. In this study, a population of EVs released by kiwi pollen during in vitro germination was isolated and characterized using different proxies. These vesicles appeared to be consistent with the pollensomes described by Prado and colleagues (Prado et al., 2014; Prado et al., 2015), and also met several criteria in the definition of small EVs and exosomes (Suhandono et al., 2014; Théry et al., 2018; Kurian et al., 2021). In fact, they were isolated by centrifugation at 100000 × g, had an average diameter under 150 nm, a rounded shape, and a double-layered lipidic membrane. They also carried proteins involved in endocytosis such as clathrin, ESCRT-related proteins, and dynamin, suggesting an endocytic origin. This thesis is supported by the presence in their proteome of proteins that are usually unconventionally secreted, and the absence of the TGN marker ARF1, hinting that they followed an unconventional secretion route. Several proteins and protein families known to be common in exosomes were found, including Ras-related Rab proteins, one of which has been proposed as a possible plant exosome marker (Regente et al., 2009). Moreover, immunoblotting revealed an enrichment in a plant homolog of a well-known mammalian EVs marker, ALIX, that is proven to be involved in ILVs formation and cargo sorting. Immunolocalization revealed the presence of ALIX homologs along the growing pollen tube, and they were associated with vesicle-like organelles localized near the pollen tube wall. However, it was not possible to visualize MVBs in electron microscopy, hence it is still difficult to assess the exact biogenesis of these EVs.

On the other hand, the present study suggests that hydration in humid atmosphere does not seem to promote EVs secretion, averting the possibility for these small vesicles to represent airborne carriers of pollen allergens in humid environmental conditions.

While further investigation into these vesicles is surely needed to confirm their biogenesis and secretion route, this work contributes to deepen the still scarce knowledge on pollen EVs, and on the proteins that are possibly involved in their formation, release, and biological function.

## Data availability statement

The data presented in this study are deposited in the PRIDE repository, accession number PXD039037.

## Author contributions

EAMV and SDD conceived the presented idea and supervised the project. CS and IA developed the theory. CS planned and performed the experiments and wrote the manuscript. CC performed the proteomic analysis and analyzed the proteomic data. DJB supervised proteomic analysis and uploaded proteomic data to PRIDE. LP, ET, and MPS participated in performing the experiments and validating the results. Atomic force microscopy was carried out by EF, while GC and CF performed the electron microscopy. All authors contributed to the manuscript critique.
