# Supplementary material for: Small extracellular vesicles released from germinated kiwi pollen (pollensomes) present characteristics similar to mammalian exosomes and carry a plant homolog of ALIX
Source: Front Plant Sci. 2023 Jan 25;14:1090026. doi: 10.3389/fpls.2023.1090026 (PMC9905850; doi:10.3389/fpls.2023.1090026)
Supplement: Supplementary file 1 [file DataSheet_1.docx]

***Supplementary Material***

**Supplementary Figure 1** Blank controls for the AFM experiment. A: particles micrographed in a sample of kiwi pollen germination medium, captured in height mode (A1), peak force error mode (A2), and 3D image mode (A3). B: particles micrographed in a sample of particle-free PBS, in height mode (B1) and in peak force error (B2). Particle height of both controls ranges between 2 and 0.5, and they tend to merge with the background.


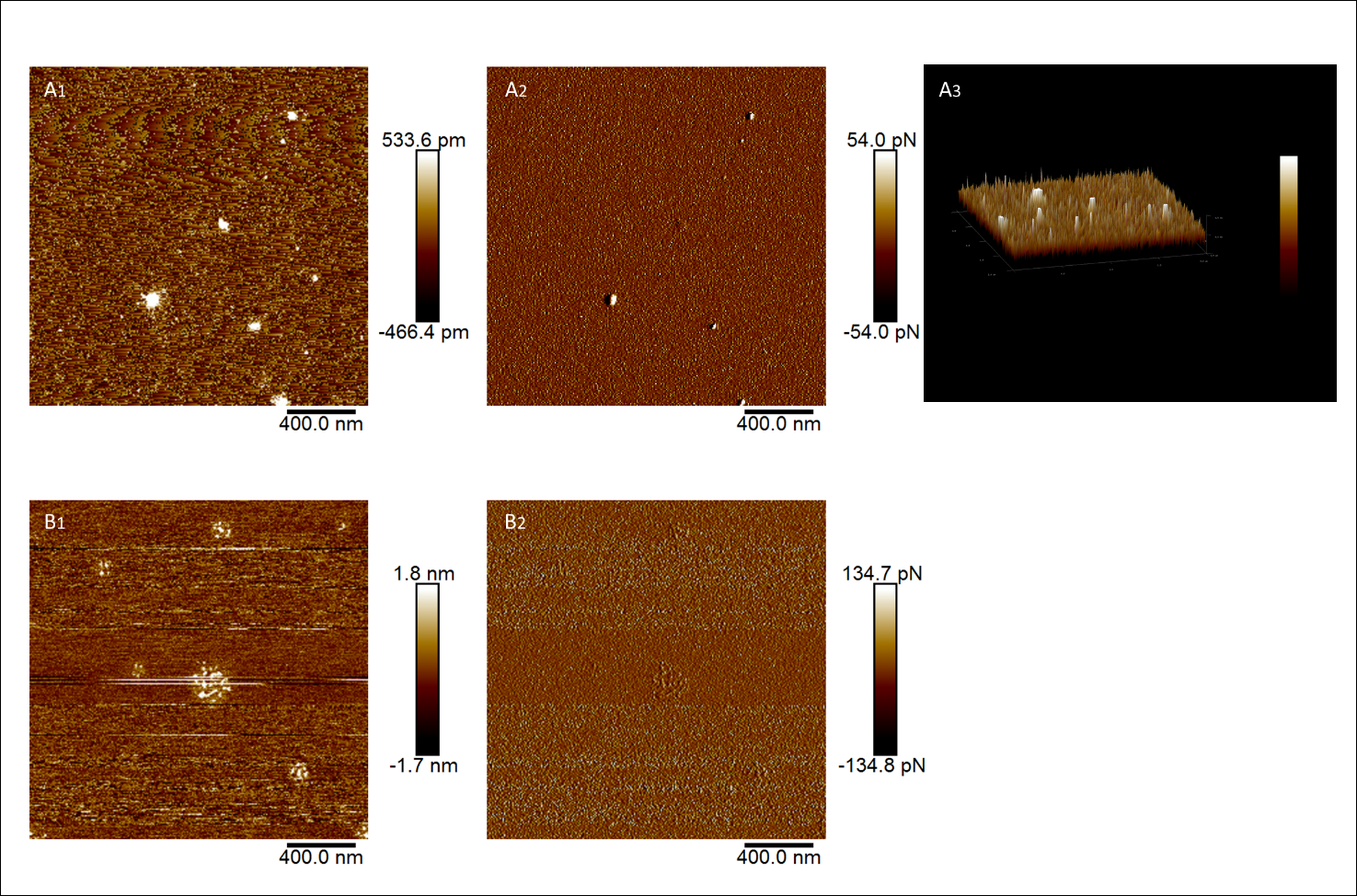


**Supplementary Figure 2** Blank controls for the FM4-64^®^ staining. A: germination medium stained with FM4-64^®^; B: particle-free PBS stained with FM4-64^®^. No fluorescent signal is detectable.
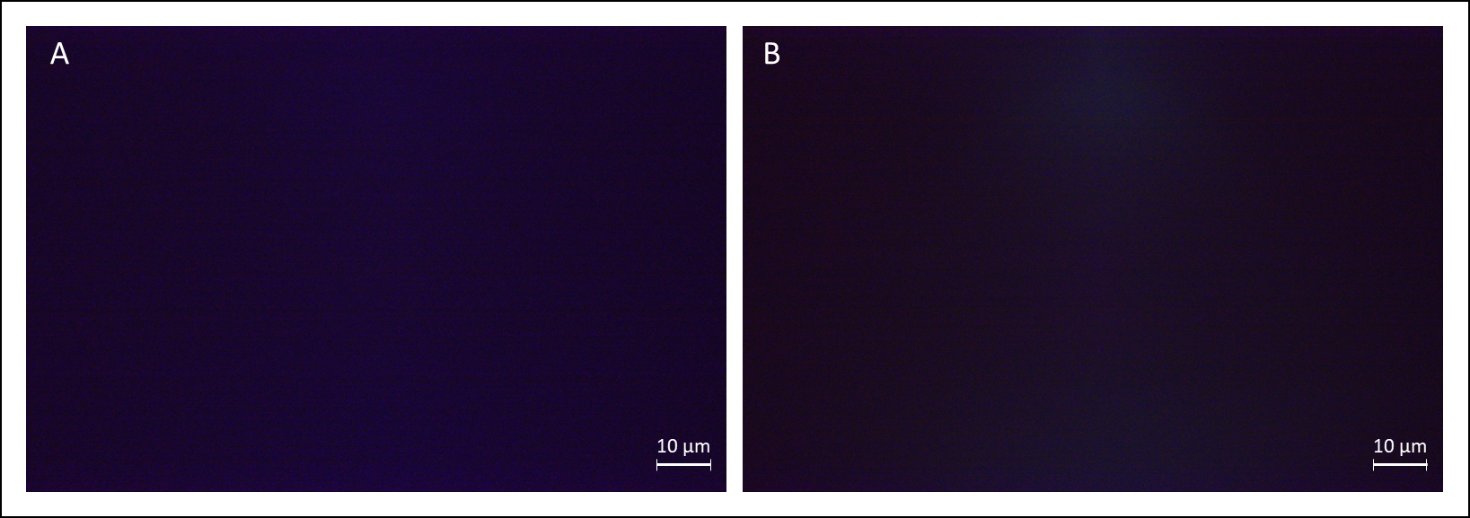


**Supplementary Figure 3** Ponceau staining of proteins from total lysate (TL), extracellular vesicles-free supernatant (EVF), and extracellular vesicles (EVs) fractions of hydrated (HKP) and germinated (GKP) kiwi pollen samples. Protein bands are not visible for EVF and EVs fractions of HKP, while they are present in all three fractions of GKP.
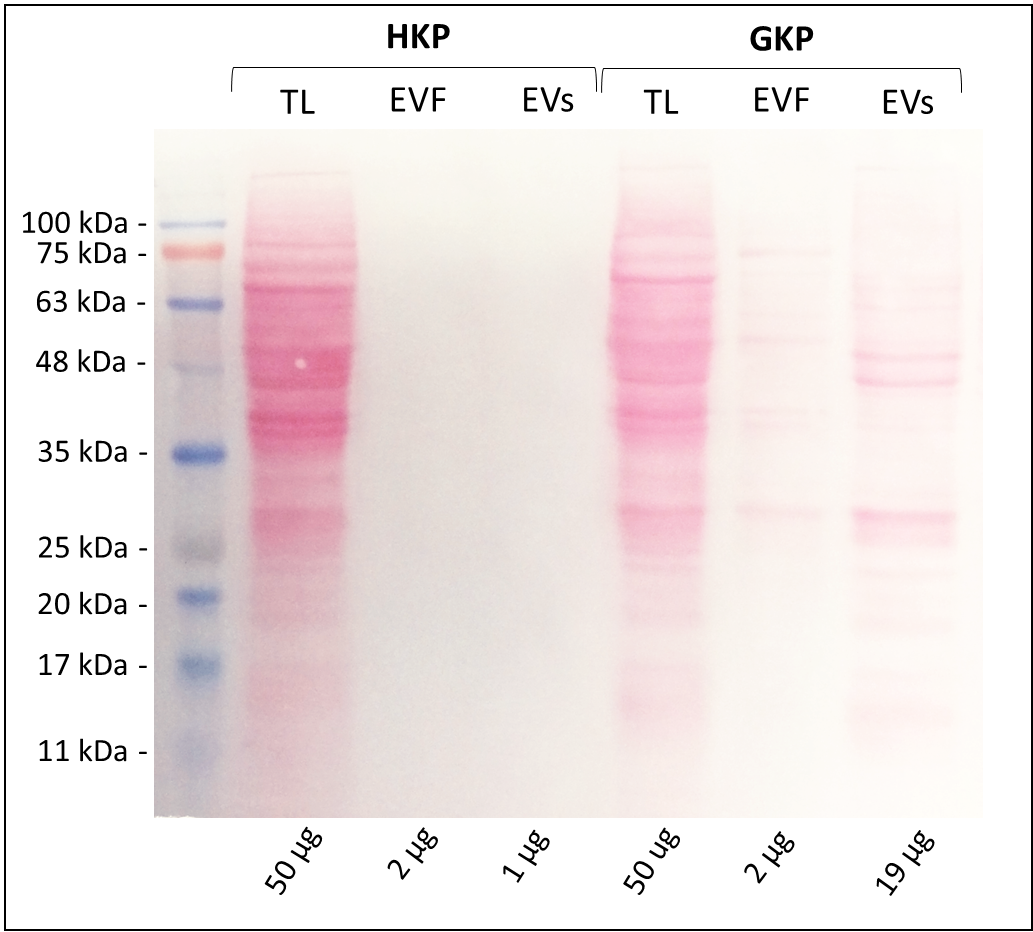


**Supplementary Figure 4** Protein-protein interactions between the 97 accession numbers identified in extracellular nanovesicles from germinated pollen of *Actinidia chinensis* Planch. with the highest accuracy (identified in all triplicates, with at least 2 peptides, over the 70% length of the peptides). The image was elaborated by STRING version 11.5. Edges thickness is proportional to the confidence associated to the interaction; darker edges indicate a higher confidence in the assessment of the interaction. Nodes without interactions are not shown. *proteins involved in carbohydrates metabolism; **26S proteasome regulatory subunits; ***proteasome subunits.



**Supplementary Figure 5** Gene Ontology (GO) terms for Biological Process, relative to the 97 selected accession numbers identified in extracellular nanovesicles from germinated pollen of *Actinidia chinensis* Planch. GO terms were provided by Panther Classification System version 17.0. The diameter of the bubbles indicates the overrepresentation of the term when compared to the whole *Actinidia chinensis* Planch. genome (Fold enrichment), while the color indicates the False Detection Rate (FDR) value. GO terms with FDR>0.05 are not shown.
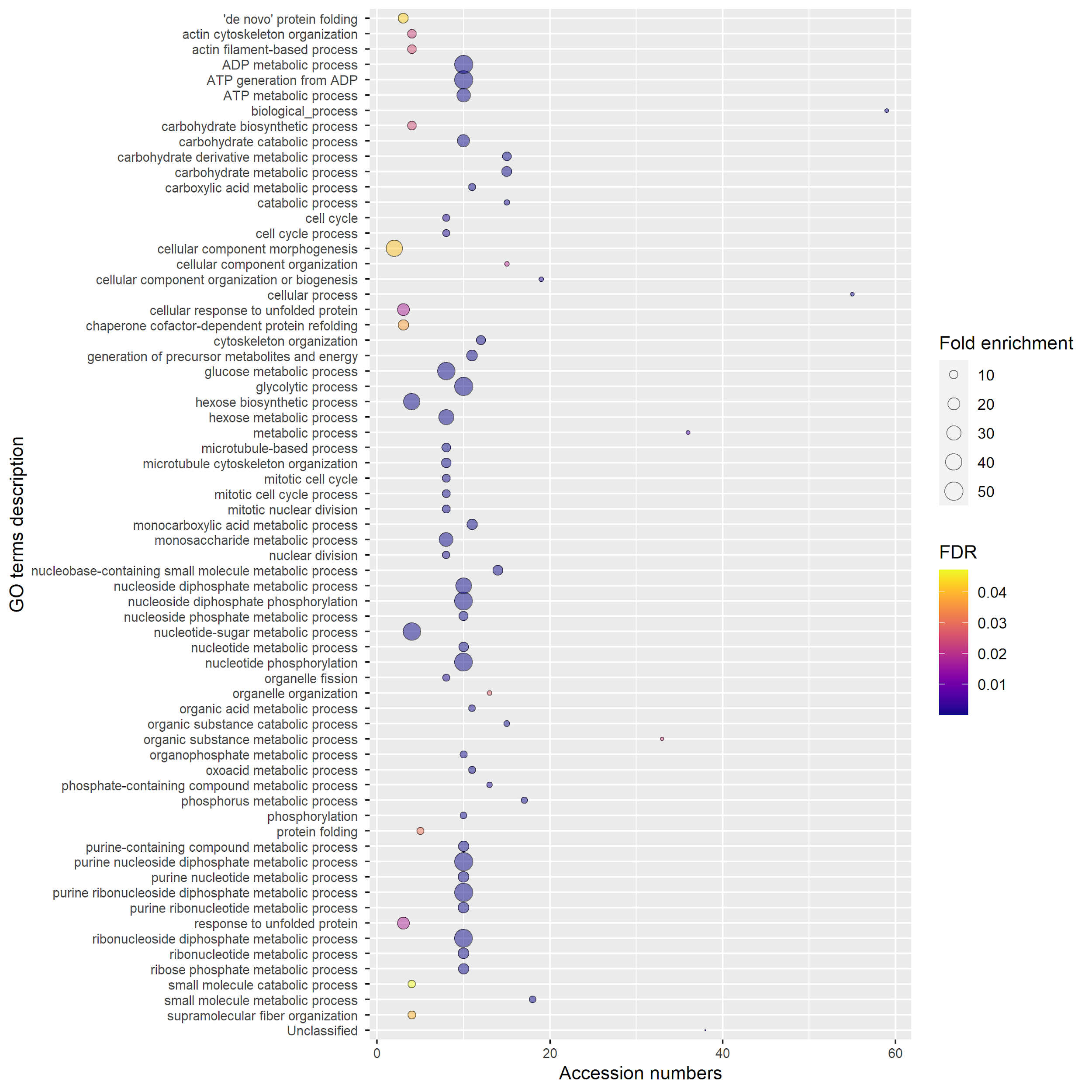


**Supplementary Figure 6** Gene Ontology (GO) terms for Molecular Function, relative to the 97 selected accession numbers identified in extracellular nanovesicles from germinated pollen of *Actinidia chinensis* Planch. GO terms were provided by Panther Classification System version 17.0. The diameter of the bubbles indicates the overrepresentation of the term when compared to the whole *Actinidia chinensis* Planch. genome (Fold enrichment), while the color indicates the False Detection Rate (FDR) value. GO terms with FDR>0.05 are not shown.


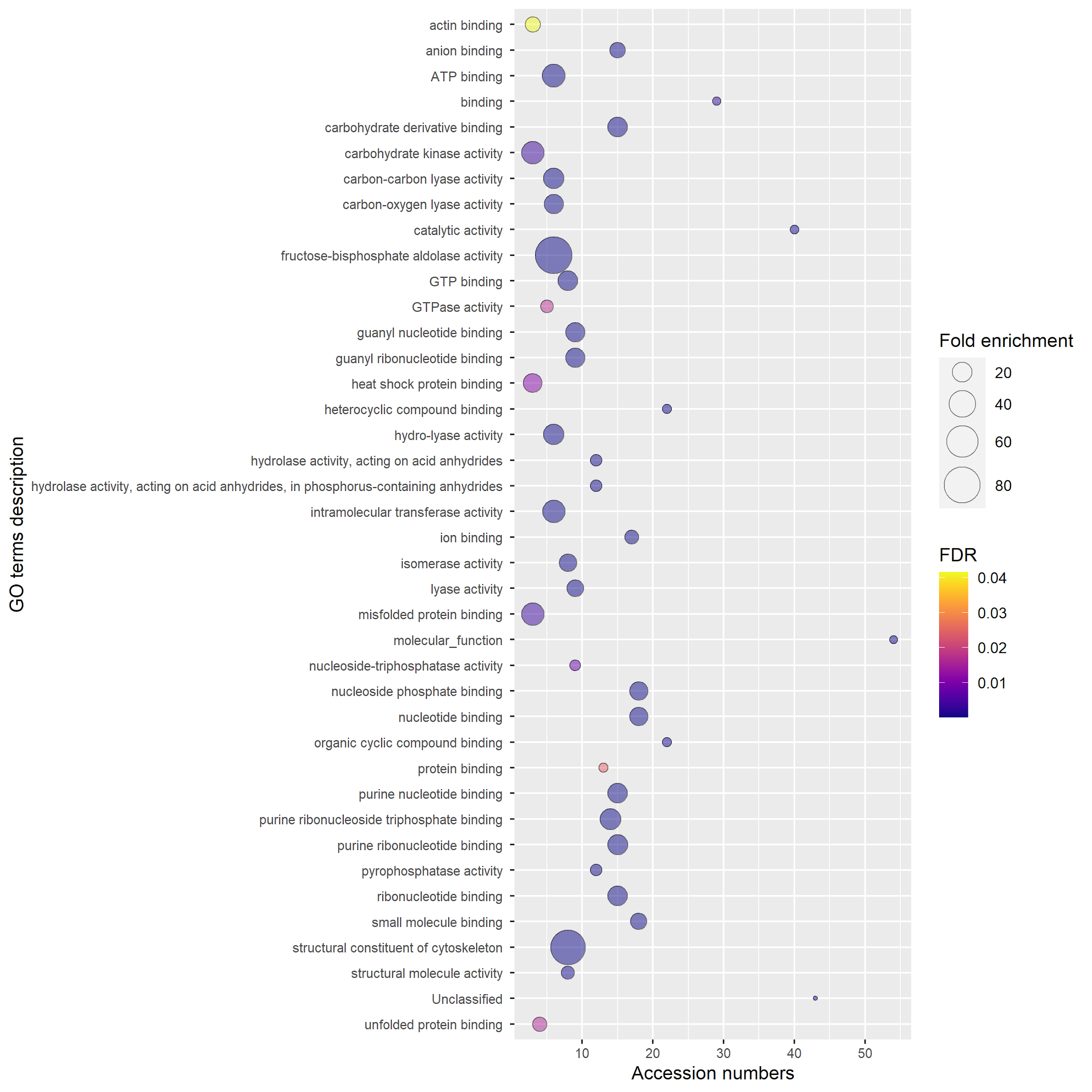


**Supplementary Figure 7** Gene Ontology (GO) terms for Cellular Component, relative to the 97 selected accession numbers identified in extracellular nanovesicles from germinated pollen of *Actinidia chinensis* Planch. GO terms were provided by Panther Classification System version 17.0. The diameter of the bubbles indicates the overrepresentation of the term when compared to the whole *Actinidia chinensis* Planch. genome (Fold enrichment), while the color indicates the False Detection Rate (FDR) value. GO terms with FDR>0.05 are not shown.
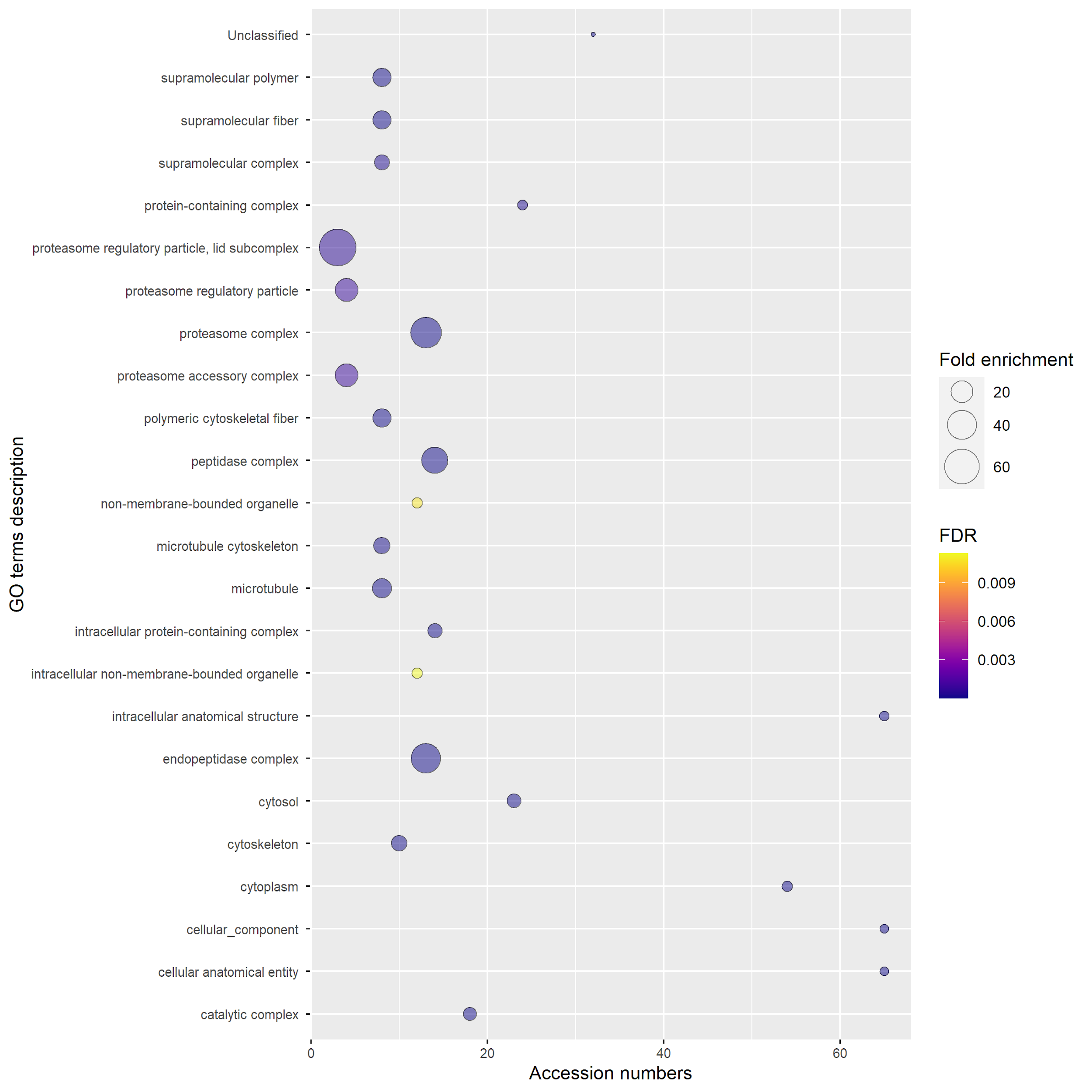


**Supplementary Figure 8** Negative controls of immunofluorescence labeling with FITC-conjugated antibodies (A) and of immunogold labeling (B), processed without anti-ALIX primary antibodies. No nonspecific signal is visible.
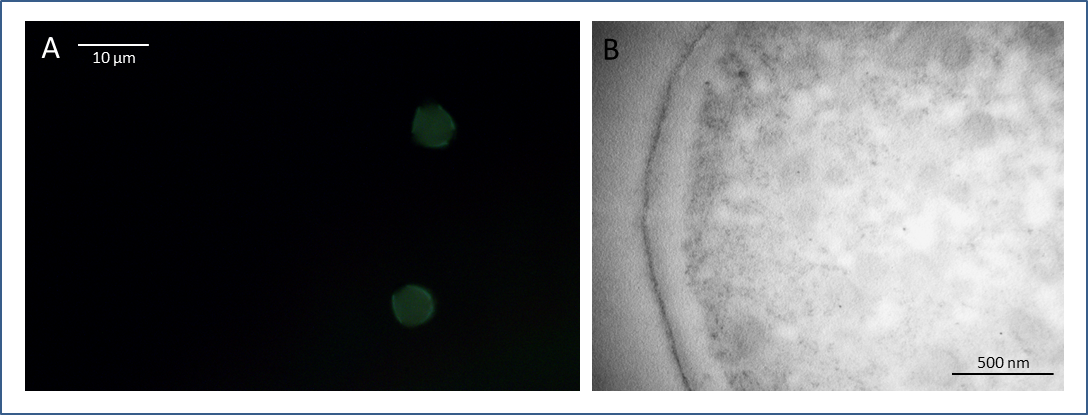


**Supplementary Table 1** Proteins identified by RP-HPLC-ESI-MS/MS in nanovesicles isolated from germinated kiwi (*Actinidia chinensis* Planch.) pollen using UniProt database, ordered by accession number. Samples were analyzed in triplicate. Count of samples= number of samples in which the protein was present; Peptides (95%) = number of peptides (95% confidence) used to identify the protein; %Cov(95)= percent length coverage with 95% confidence.

*Supplementary Table 1 is available at the following link:* <https://osf.io/sm378/?view_only=6cc3bb794fb84eb6a1f18da44605826c>

**Supplementary Table 2** List of the proteins identified in all three replicas of germinated kiwi pollen EV samples, identified using 2 or more peptides and with 95% coverage over 70% of the length of the peptides. Proteins are ordered by decreasing values of maximum length of 95% coverage and maximum number of peptides. Information not available on UniProtKB and/or in literature was marked as “Unknown”.

| Protein | Family | Cellular localisation | Biological process |
| --- | --- | --- | --- |
| Late embryogenesis abundant protein | Late embryogenesis abundant protein (LEA) family protein (90% homology) | Plasma membrane | Response to dehydration |
| Fructose-bisphosphate aldolase | Class I fructose-bisphosphate aldolase family | Cytoplasm, plastids | Glycolytic process |
| Stress-induced protein | Late embryogenesis abundant protein (LEA) family protein (90% homology) | Unknown | Stress response |
| S-adenosylmethionine synthase | Adomet synthase family | Cytoplasm | One-carbon metabolic process,  s-adenosylmethionine biosynthetic process |
| UDP-arabinopyranose mutase | Reversibly Glycosylated Proteins (RGP) family | Cytoplasm | Plant-type cell wall organization or biogenesis |
| Proteasome subunit alpha type (Fragment) | Peptidase T1A family | Cytoplasm, nucleus | Ubiquitin-dependent protein catabolic process |
| Carbonic anhydrase | Beta-class carbonic anhydrase family | Cytoplasm, plastids | Carbon utilization |
| Adenosylhomocysteinase | Adenosylhomocysteinase family | Cytoplasm, plasma membrane, vacuole, tonoplast | One-carbon metabolic process |
| Adenylate kinase (Fragment) | Adenylate kinase family | Cytoplasm, plastids | ‘De novo’ pyrimidine nucleobase biosynthetic process |
| Inorganic diphosphatase | Ppase family | Cytoplasm | Phosphate-containing compound metabolic process |
| Calcium-binding protein | EF hand calcium-binding protein family (90% homology) | Unknown | Calcium ion binding |
| Proteasome subunit beta | Peptidase T1B family | Cytoplasm, nucleus | Proteolysis involved in cellular protein catabolic process |
| 26S proteasome non-ATPase regulatory subunit 8 A like | Proteasome subunit S14 family | Proteasome, membrane | Proteolysis |
| Phosphoglycerate kinase | Phosphoglycerate kinase family | Chloroplast, cytoplasm, nucleus | Glycolytic process |
| UTP--glucose-1-phosphate uridylyltransferase | UDPGP type 1 family (50% similarity) | Unknown | Uridylyltransferase activity |
| UDP-glucuronate decarboxylase | NAD(P)-dependent epimerase/dehydratase family UDP-glucuronic acid decarboxylase subfamily | Membrane | D-xylose metabolic process, UDP-d-xylose biosynthetic process |
| Proteasome subunit alpha type | Peptidase T1A family | Nucleus, cytoplasm | Ubiquitin-dependent protein catabolic process |
| Triosephosphate isomerase | Triosephosphate isomerase family | Cytoplasm, plastids | Glycolytic process |
| Phosphopyruvate hydratase | Enolase family | Cytoplasm, nucleus | Glycolytic process |
| 26S protease regulatory subunit 6A (Fragment) | AAA ATPase family | Cytoplasm, proteasome | Protein catabolic process |
| Phosphoglucomutase (alpha-D-glucose-1,6-bisphosphate-dependent) | Phosphohexose mutase family | Cytoplasm, plastids | Glucose metabolic process |
| Glyceraldehyde-3-phosphate dehydrogenase | Glyceraldehyde-3-phosphate dehydrogenase family | Cytoplasm, plastids | Glucose metabolic process |
| Tubulin beta chain | Tubulin family | Cytoskeleton | Microtubule-based process |
| Eukaryotic initiation factor 4A-14 | Eukaryotic initiation factor 4A-14 family | Unknown | Protein biosynthesis |
| Actin-depolymerizing factor 7 like (Fragment) | Actin-binding proteins ADF family | Cytoskeleton | Actin filament depolymerization |
| Profilin | Profilin family | Cytoskeleton | Actin-binding |
| Glutaredoxin like | Glutaredoxin family CPYC subfamily | Unknown | Electron transport |
| 26S protease regulatory subunit 6A like | AAA ATPase family | Cytoplasm, proteasome | Protein catabolic process |
| Heat shock protein 70 family protein | Heat shock protein 70 family | Cytoplasm, nucleus, mitochondria | Stress response |
| UDP-glucose 6-dehydrogenase | UDP-glucose/GDP-mannose dehydrogenase family | Cell wall, cytoplasm, nucleus, secretory vesicles | UDP-glucuronate biosynthetic process |
| Guanosine nucleotide diphosphate dissociation inhibitor | Rab GDI family | Cytoplasm, apoplast, plasmodesma | Protein transport, small GTPase mediated signal transduction |
| Mediator of RNA polymerase II transcription subunit 37e | Heat shock protein 70 family | Unknown | ATPase activity |
| UDP-4-keto-6-deoxy-D-glucose 3,5-epimerase/UDP-4-keto-L-rhamnose 4-keto-reductase | Unknown | Nucleus, cytoplasm, plasmodesma | Nucleotide-sugar metabolic process |
| Elongation factor 1-alpha | TRAFAC class translation factor GTPase superfamily Classic translation factor GTPase family, EF-Tu/EF-1A subfamily | Cytoplasm | Unknown |
| Actin-97 | Actin family | Cytoskeleton | ATP-binding |
| Pyrophosphate--fructose 6-phosphate 1-phosphotransferase subunit alpha | Phosphofructokinase type A (PFKA) family,  ppi-dependent PFK group II subfamily Clade 'Long' sub-subfamily | Cytoplasm | Glycolytic process |
| Actin-7 | Actin family | Cytoskeleton | ATP-binding |
| Pyrophosphate--fructose 6-phosphate 1-phosphotransferase subunit beta | Phosphofructokinase type A (PFKA) family, ppi-dependent PFK group II subfamily Clade 'Long' sub-subfamily | Cytoplasm | Fructose 6-phosphate metabolic process (glycolytic process) |
| 14-3-3-like protein | 14-3-3 family | Unknown | Unknown |
| Glycine-rich RNA-binding protein | Unknown | Unknown | RNA-bidning |
| Bifunctional dTDP-4-dehydrorhamnose 3,5-epimerase/dTDP-4-dehydrorhamnose reductase | dTDP-4-dehydrorhamnose reductase family | Cytoplasm, plasma membrane, plasmodesma | Cell wall organization, dTDP-rhamnose biosynthetic process, UDP-rhamnose biosynthetic process |
| 26S proteasome non-ATPase regulatory subunit like | Proteasome subunit S9 family  Proteasome subunit S5A family | Proteasome | Protein catabolic process |
| Peptidyl-prolyl cis-trans isomerase | Cyclophilin-type ppiase family | Unknown | Protein folding |
| Fructokinase-4 like | Carbohydrate kinase pfkb family | Unknown | Starch biosynthetic process |
| Acetyl-CoA acetyltransferase | Thiolase family | Cytoplasm | Acyltransferase |
| Elongation factor (Fragment) | Classic translation factor GTPase family, EF-G/EF-2 subfamily | Cytoplasm | Protein biosynthesis |
| CCT-beta (Fragment) | TCP-1 chaperonin family | Unknown | Protein folding |
| Clathrin heavy chain like | Clathrin heavy chain family | TGN, plasma membrane | Intracellular protein transport, vesicle-mediated transport |
| Calmodulin | Unknown | Unknown | Calcium ion binding |
| 26S protease regulatory subunit 7 | AAA ATPase family | Cytoplasm, proteasome | Protein catabolic process |
| 26S protease regulatory subunit 7B | AAA ATPase family | Cytoplasm, proteasome | Protein catabolic process |
